# Inhibition and Larvicidal Activity of Phenylpropanoids from *Piper sarmentosum* on Acetylcholinesterase against Mosquito Vectors and Their Binding Mode of Interaction

**DOI:** 10.1371/journal.pone.0155265

**Published:** 2016-05-06

**Authors:** Arshia Hematpoor, Sook Yee Liew, Wei Lim Chong, Mohd Sofian Azirun, Vannajan Sanghiran Lee, Khalijah Awang

**Affiliations:** 1 Institute of Biological Sciences, Faculty of Science Building, University of Malaya, Kuala Lumpur, Malaysia; 2 Department of Chemistry, Faculty of science, University of Malaya, Kuala Lumpur, Malaysia; 3 Centre for Natural Products and Drug Discovery (CENAR), University of Malaya, Kuala Lumpur, Malaysia; Institute of Vegetables and Flowers, Chinese Academy of Agricultural Sciences, CHINA

## Abstract

*Aedes aegypti*, *Aedes albopictus* and *Culex quinquefasciatus* are vectors of dengue fever and West Nile virus diseases. This study was conducted to determine the toxicity, mechanism of action and the binding interaction of three active phenylpropanoids from *Piper sarmentosum* (Piperaceae) toward late 3^rd^ or early 4^th^ larvae of above vectors. A bioassay guided-fractionation on the hexane extract from the roots of *Piper sarmentosum* led to the isolation and identification of three active phenylpropanoids; asaricin **1**, isoasarone **2** and *trans*-asarone **3**. The current study involved evaluation of the toxicity and acetylcholinesterase (AChE) inhibition of these compounds against *Aedes aegypti*, *Aedes albopictus* and *Culex quinquefasciatus* larvae. Asaricin **1** and isoasarone **2** were highly potent against *Aedes aegypti*, *Aedes albopictus* and *Culex quinquefasciatus* larvae causing up to 100% mortality at ≤ 15 μg/mL concentration. The ovicidal activity of asaricin **1**, isoasarone **2** and *trans*-asarone **3** were evaluated through egg hatching. Asaricin **1** and isoasarone **2** showed potent ovicidal activity. Ovicidal activity for both compounds was up to 95% at 25μg/mL. Asaricin **1** and isoasarone **2** showed strong inhibition on acetylcholinesterase with relative IC_50_ values of 0.73 to 1.87 μg/mL respectively. These findings coupled with the high AChE inhibition may suggest that asaricin **1** and isoasarone **2** are neuron toxic compounds toward *Aedes aegypti*, *Aedes albopictus* and *Culex quinquefasciatus*. Further computational docking with Autodock Vina elaborates the possible interaction of asaricin **1** and isoasarone **2** with three possible binding sites of AChE which includes catalytic triads (CAS: S238, E367, H480), the peripheral sites (PAS: E72, W271) and anionic binding site (W83). The binding affinity of asaricin **1** and isoasarone **2** were relatively strong with asaricin **1** showed a higher binding affinity in the anionic pocket.

## Introduction

Between mosquito species, *Aedes aegypti*, *Aedes albopictus* and *Culex quinquefasciatus*, are among the important vectors of wide range of diseases such as dengue fever and West Nile virus. These species are cosmopolitan in distribution and commonly found in tropical and subtropical countries including Malaysia [1–4]. With growing usage of synthetic insecticides, there have been reports on mosquito resistance to organophosphates which resulted from consistent spraying and application [3, 5]. In the process of transition of impulses between the neurons, acetylcholinesterase (AChE) is a key enzyme, which hydrolyses the neurotransmitter acetylcholine (ACh) in cholinergic synapses of neurons [6]. Therefore, AChE plays a crucial act for every function of living being including insects. Most of current insecticides such as organophosphorus (OP) is based on inhibiting acetylcholinesterase (AChE), yet the knowledge of this enzyme and its mechanism of action is limited [7–9]. AChE catalyses the hydrolysis of acetylcholine, a neurotransmitter for cholinergic neurotransmission in insects. The neurotoxic compounds hydrolyse the neurotransmitter acetylcholine (ACh) to terminate neuronal excitement at the postsynaptic membrane [10]. In mosquitoes, resistance is usually caused by less inhibition of the target enzyme in the resistant strains [11, 12]. Better understanding of the pesticide action on enzymes in insect pests should enable to produce selective and effective insecticides with low mammalian toxicity [7, 13]. Finding new biopesticides and knowing the mechanism of action will help to control the resistance in insects’ pests, which is an increasing problem in insect control [14].

Botanical products have been in the centre of interest as sources of secondary metabolites for insecticide discovery. *Piper sarmentosum* is a member of Piper family, widely distributed in tropical and sub-tropical regions of the world. It is a glabrous, creeping terrestrial herb of about 20 cm in height, and because of aromatic odour and a pungent taste, it has been used in local cuisine [15]. Plants of the Piper family have been traditionally used in folk medicine as treatment of diabetes mellitus, cough, toothache, fungal infection on the skin [15, 16] and several species have shown insecticidal activity such as *Piper retrofractum* and *Piper nigrum* L [17, 18]. *P*. *sarmentosum* extracts are rich source of biological and chemical diversity including phenylpropanoids [19]. These compounds are detected in essential oil and plant extracts which are known to have larvicidal activity against mosquitoes and several other compounds have been isolated with AChE inhibition activity [20–22]. Hence, these prior findings led us to investigate the larvicidal activity of active components from *P*. *sarmentosum* and their mechanism of action on AChE of *Ae*. *aegypti*, *Ae*. *albopictus* and *Cx*. *quinquefasciatus*. Subsequently, a molecular docking study was undertaken to enlighten further the possible molecular interactions between the active compounds and the enzyme acetylcholinesterase.

## Materials and Methods

### General experimental procedures

Column chromatography was performed using silica gel 60, 230–400 mesh ASTM (Merck, 0.040–0.063mm). Aluminium supported silica gel 60 F_254_ plates 20 x 20cm (absorbent thickness: 0.25 mm) were used for thin layer chromatography (TLC) (Merck, Germany). Preparative thin layer chromatography (PTLC) (Merck, Germany) silica gel 60 F_254_ glass plates 20 x 20cm (absorbent thickness: 0.50 mm) were used for separation of compounds. IR spectrum was recorded using a Perkin-Elmer Spectrum 400 FT-IR Spectrometer with spectroscopic grade chloroform as the solvent. 1D- and 2D-NMR spectra were recorded in chloroform CDCl_3_ (Merck, Germany) using JEOL ECA 400 MHz NMR spectrometer. The LCMS-IT-TOF spectra were recorded on a UFLC Shimadzu Liquid Chromatography with a SPD-M20A diode array detector coupled to a IT-TOF mass spectrometer. UV spectra were recorded using a Shimadzu 1650 PC UV-Vis Spectrophotometer with spectroscopic grade methanol (CH_3_OH) as solvent. All solvents were of analytical grade and were distilled prior to use.

### Plant material

*P*. *sarmentosum* was collected in the vicinity of University of Malaya in 2011 and identified by Mr. Teo Leong Eng. A voucher specimen (KU 0110) was deposited at the Herbarium of the Department of Chemistry, University of Malaya, Kuala Lumpur, Malaysia.

### Extraction and Isolation

**Fig 1:**
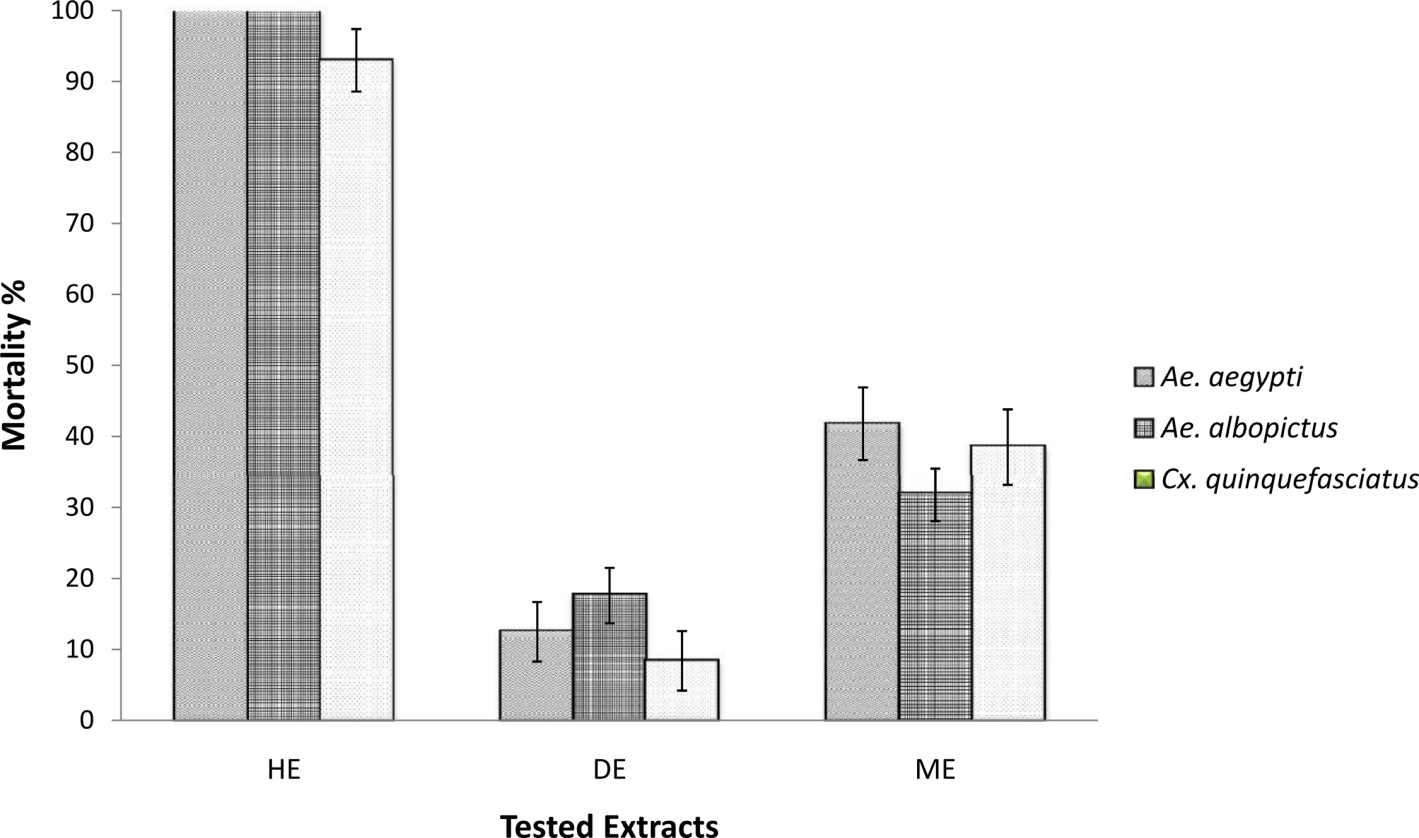
Preliminary larvicidal activity on extracts. Roots methanol extracts (ME), hexane extracts (HE), dichloromethane extracts (DE) were tested on *Ae*. *aegypti*, *Ae*. *albopictus* and *Cx*. *quinquefasciatus* late third early fourth instar larvae.

**Fig 2:**
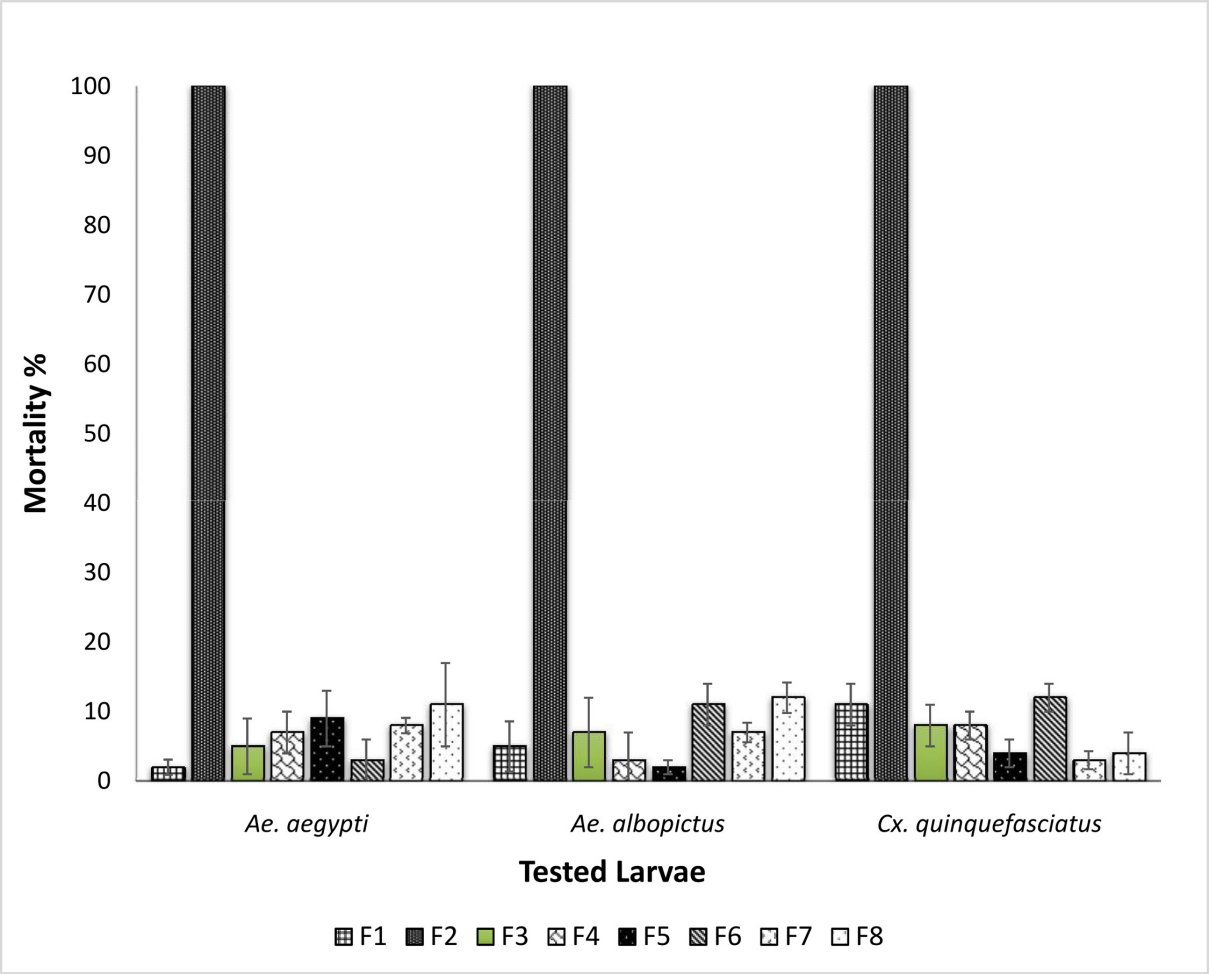
Preliminary larvicidal activity on fractions. All fractions (F1-F8) from roots hexane extracts tested on *Ae*. *aegypti*, *Ae*. *albopictus* and *Cx*. *quinquefasciatus* late third early fourth instar larvae.

**Fig 3:**
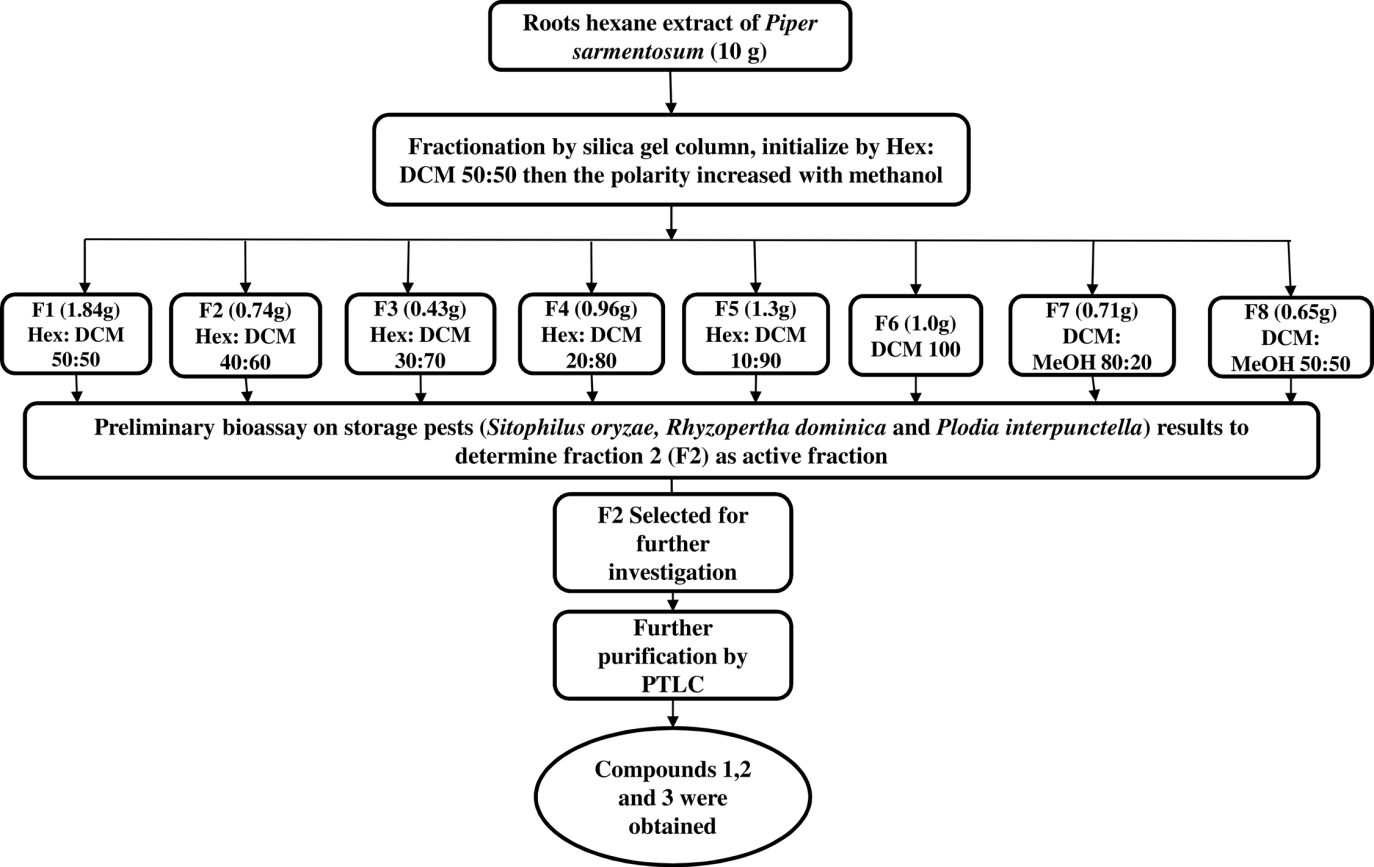
Isolation procedures of asaricin 1, isoasarone 2 and *trans*-asarone 3 as larvicide. The *P*. *sarmentosum* roots hexane extract was fractionated using hexane- dichloromethane. The preliminary test had identify the active fraction and relative active fraction (F2) were further purified using thin layer chromatography (TLC). Isolated asaricin **1**, isoasarone **2** and *trans*-asarone **3** were tested toward late third early fourth instar larvae from *Ae*. *aegypti*, *Ae*. *albopictus* and *Cx*. *quinquefasciatus*.

Dried and powdered roots (1 kg) of *P*. *sarmentosum* were extracted successively twice with hexane (3 L), dichloromethane (3 L) followed by methanol (3 L) at room temperature, giving 9.79 g, 23.08 g and 16.42 g of extracts, respectively. In a preliminary screening of the potential toxicity of the extracts towards *Ae*. *aegypti*, *Ae*. *albopictus* and *Cx*. *quinquefasciatus*, the highest percentage mortality was observed for the hexane extract (Fig 1). Therefore, this extract was subjected to bioassay guided fractionation. The hexane extract was subjected to column chromatography over silica gel by eluting with hexane gradually enriched with CH_2_Cl_2_ (0–100%) followed by CH_2_Cl_2_ gradually enriched with MeOH (0–100%). Out of the eight fractions obtained (F1-F8), fraction F2 (0.47 g) was found to exhibit the highest toxicity towards *Ae*. *aegypti*, *Ae*. *albopictus* and *Cx*. *quinquefasciatus* (Fig 2). This fraction was further purified with preparative TLC using hexane:CH_2_Cl_2_ (50:50 v/v) which led to the isolation of asaricin **1** (67 mg) (R_f_ = 0.92), isoasarone **2** (40 mg) (R_f_ = 0.66) and *trans*-asarone **3** (180 mg) (R_f_ = 0.64) (Fig 3).

### Characterization of isolated compounds

**Fig 4:**
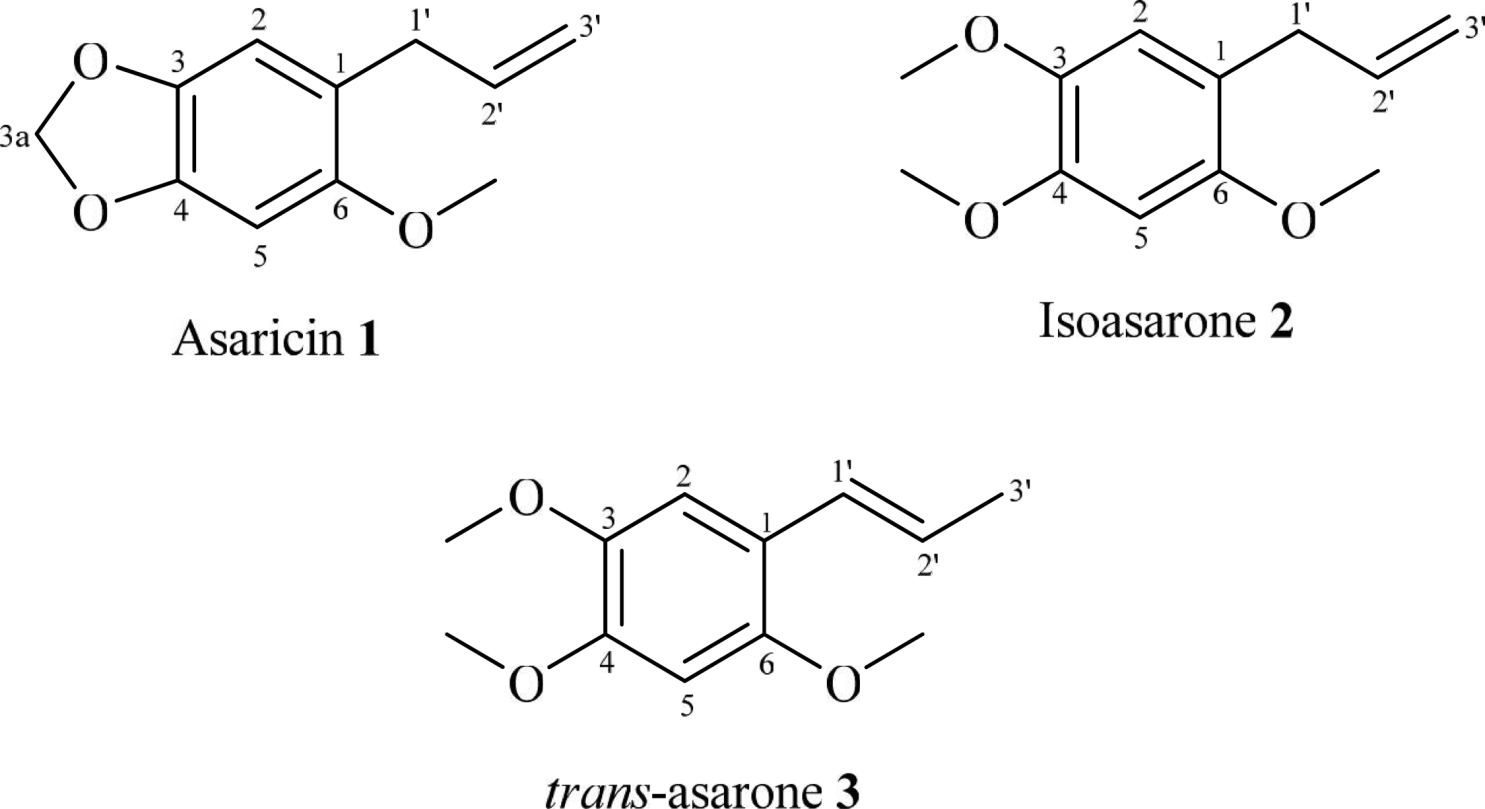
Chemical constituents; asaricin 1, isoasarone 2 and *trans*-asarone 3 which were isolated from roots of *P*. *sarmentosum*.

Bioassay guided fractionation and isolation of the hexane extract led to the isolation of three phenylpropanoids **1–3**. The structures of the compounds (Fig 4) were identified as asaricin **1,** isoasarone **2** and *trans*-asarone **3** with the aid of various spectroscopic methods such as 1D-NMR (^1^H, ^13^C, DEPT), 2D-NMR (COSY, HMBC, HSQC), LCMS, UV and IR as well as comparison with those reported in the literature [23–25].

### Insecticidal activity

#### Test Mosquito

All tested insects used for the experiments were obtained from colonies cultured in the laboratory at 27 ± 2°C and 65 ± 5% relative humidity. To determine the susceptibility, three species of adult mosquitoes and larvae of *Ae*. *aegypti*, *Ae*. *albopictus* and *Cx*. *quinquefasciatus* were used as sample and were measured over a period of 1 year (2012–2013) in this study. Each different species was categorized and reared in laboratory as laboratory strains. *Ae*. *aegypti*, *Ae*. *albopictus* and *Cx*. *quinquefasciatus* were collected from cemetery near the Malaysia International Airport KLI, 2011. Mosquito larvae were placed in plastic containers located in cages (23cm x 23cm x 23cm) at laboratory condition with a photoperiod of 13 hours of daylight and 11 hours of darkness for emergence.

#### Larval Preliminary bioassay

The larval bioassay was performed on late 3^rd^ or early 4^th^ larvae of *Ae*. *aegypti*, *Ae*. *albopictus* and *Cx*. *quinquefasciatus* according to the WHO larvicidal activity standard procedure [26]. 15 mg of asaricin **1,** isoasarone **2** and *trans*-asarone **3** were separately dissolved in 1 mL of acetone as stock solution and several concentrations (4 to 20 μg/mL) were prepared through serial dilution by mixing specific amount of stock solution with 250mL distilled water. Twenty five larvae of *Ae*. *aegypti*, *Ae*. *albopictus* and *Cx*. *quinquefasciatus* were separately introduced into the plastic cups containing appropriate concentrations of asaricin **1,** isoasarone **2** and *trans*-asarone **3**. Control was only treated with 1 mL acetone added to 250mL distilled water. Mortality of the tested larvae was recorded at the end of 24 hours. Each treatment was replicated four times.

$$\%\text{larvae mortality}=\frac{\text{Number of dead larvae}}{\text{Total number of introduced larvae}}\times 100\%$$

#### Ovicidal Activity

Ovicidal activity was assessed by modifying the method of Kamaraj and co-worker [27]. The eggs of *Cx*. *quinquefasciatus*, *Ae*.*aegypti* and *Ae*. *albopictus* were collected from the laboratory batch. Pure compounds; asaricin **1,** isoasarone **2** and *trans*-asarone **3**, were separately dissolved in 1 mL acetone and further diluted with distilled water to achieve various concentrations ranging from 5 to 25 μg/ml inside the disposable testing plastic cups with 250 mL capacity. 25 eggs of each tested mosquito species were individually exposed to each concentration in the cups. The tests for each concentration were performed in 4 replicates. After 48 hours of treatment, the number of dead eggs from each concentration were individually recorded under microscope. The percentage of ovicidal activity was calculated by using the following formula:

$$\%\;\text{of egg mortality}=\frac{\text{Number of unhatched eggs}}{\text{Total number of eggs}}\times 100\%$$

#### Repellent activity

This study was carried out according to the method described by WHO standard procedure [28]. Three-day-old blood-starved female of *Ae*.*aegypti*, *Ae*. *albopictus* and *Cx*. *quinquefasciatus* (100 each) were kept in a net cage (23 cm × 23 cm × 23 cm). Hands that had no contact with lotions, perfumes or perfumed soaps were exposed to the mosquito cage. Only 25 cm^2^ dorsal side of the skin on each arm was exposed and the remaining area covered by rubber gloves. The tested substance was applied at several dosages, separately in the exposed area of the forearm. Acetone was served as control. The timing of the test depended on whether the tested mosquitoes were day or night biters. *Ae*.*aegypti* and *Ae*. *albopictus* were tested from 6 am to 9 am hours, while *Cx*. *quinquefasciatus* was tested from 8 am to 7 pm hours. Both control and treated arms were introduced simultaneously into the mosquito cage, and followed by gentle tapping on the sides of the experimental cages to activate the mosquitoes. Each test concentration was repeated four times. The volunteers conducted test for each concentration by inserting the treated and control arms into the same cage for one minute and repeated this action every five minutes. The mosquitoes that landed on the hand were recorded and then shaken off before imbibing any blood. The percentage of repellency was calculated by the following formula,

$$\%\;\text{Repellency}\;=\left[\frac{\text{Ta}\;-\;\text{Tb}}{\text{Ta}}\right]\times 100\%\;,\;\text{where}$$

Ta: the number of mosquitoes in the control group.

Tb: the number of mosquitoes in the treated group.

#### Enzyme preparation

For this experiments, the larvae were removed and homogenized at 0°C (keeping the homogenized mixture in plastic tube covered with ice) in 0.05 M-phosphate buffer, pH7.5 (KH_2_PO_4_-NaOH) (Merck, USA) by using a glass homogenizer with a polytetrafluoroethylene (PTFE) pestle (5%, w/v, homogenate). The homogenates were centrifuged for 20 min at 5°C and 16000 rpm. These supernatants were collected with pipette and kept inside the ice for studying the AChE inhibition biochemical assay.

### Acetylcholinesterase inhibition assay (in vitro)

To determine the IC_50_ values, procedures from Ellman et al., 1961 were modified and applied [29–31]. Electronic multichannel pipette (Eppendorf, USA) was used to transfer exact 10 μL of enzyme solution to each well of a 96-well microtiter plate further mixed with 20 μL of tested compounds in solution form and 150 μL of cold phosphate buffer which were kept in refrigerator 1–4°C. The assay microtiter plates were incubated at 25°C for 10 min. Inhibitors were dissolved in DMSO (Merck, USA) in several concentration and stocks diluted to give a final concentration of 0.1% DMSO (v/v) were added into test well in separated rows. This was followed by adding 20 μL of acetylthiocholine iodide (ACTHI) (Sigma-Aldrich, USA) (0.4 mM) and DTNB (Sigma-Aldrich, USA) (0.3 mM) to enzyme solution to observe the reaction. Yellowish or colorless solution was observed during reaction for 30 minutes at room temperature. Changes in absorbance were recorded by a microplate readers (Synergy H1 Hybrid Multi-Mode Microplate Reader, USA) at 412 nm. Enzyme concentrations used were within the linear range of their toxicity activity.

$$\%\;\text{Inhibition}\;=\left[\frac{A\text{con}\;-A\text{sam}}{A\text{con}}\right]\times 100\%\;,\;\text{where}$$

*A*con: the absorbance of the control

*A*sam: the absorbance of the test sample.

The IC_50_ was calculated by log-probit analysis using Polo Plus program [32]. Another set of supernatant was loaded with 20 μL of ACTHI and DTNB solution only which served as control. The mean IC_50_ significant values were statistically determined by comparison of confident interval.

### Molecular docking study

The initial structure of *Drosophila melanogaster* acetylcholinesterase (AChE) was retrieved from protein data bank (http://www.pdb.org) with PDB ID:1QON. The enzyme was then prepared under the protein preparation protocol implemented in Discovery Studio (Accelry Inc, 2.5.5) [33] in which missing atoms in the incomplete residues were added, alternate conformations were removed and the atom names were standardized. The missing residues from 103–135, 574–585 were not included in this model as the residues were located very far from the investigated binding sites. Prior to minimization using smart minimizer algorithm, molecular properties of both compounds and the enzyme were described by CHARMm forcefield [34] with Momany-Rone for the partial charge setting. The complex of enzyme and compound was generated using Autodock Vina [35] by allowing the ligand to be flexible. The input site sphere was set at the three binding sites a) catalytic binding site which include three catalytic triads (CAS:S238,E367,H480), b) the peripheral site (PAS:E72,W271) which is above the active site and close to the mouth of the gorge on the protein surfaced, and c) anionic binding site (W83) [36]. The docked complexes would be further minimized and calculated for their respective interaction energy per residue.

## Results

### Preliminary larvicidal assay

The toxicity potential of the hexane (HE), dichloromethane (DE) and methanol extracts (ME) from the roots of *P*. *sarmentosum* were successfully tested on *Ae*. *aegypti*, *Ae*. *albopictus and Cx*. *quinquefasciatus* larva. Based on the results obtained, the hexane extract exhibited the highest percentage of mortality for all three mosquito vectors at a concentration level of 250 μg/mL over an exposure period of 24 hours. Hence, HE was subjected to toxicity guided fractionation which resulted in 8 fractions (F1-F8). F2 was highly potent against tested larvae causing up to 100% mortality at 150 μg/mL concentration. The rest of the fractions were not potent. Potent toxicity of F2 led to the isolation and characterization of asaricin **1** and isoasarone **2** and *trans*-asarone **3** as the active constituents (Fig 3).

### Isolation and identification of insecticidal substances from *P*. *sarmentosum*

Asaricin **1** was isolated as an optically inactive yellow oil. The LCMS-IT-TOF mass spectrum revealed a pseudomolecular ion peak [M+H]^+^ at *m/z* 192.21118. The molecular mass was supported by the ^13^C NMR spectrum (Table 1) which confirmed the presence of only 11 carbon signals. The IR spectrum exhibited characteristic absorption peaks of aromatic (2925 cm^−1^) and methylenedioxy (1040 and 933 cm^−1^) stretching vibration [37].

**Table 1:** ^1^H-NMR (400 MHz) and ^13^C-NMR (100 MHz) spectral data of asaricin 1 in CDCl_3_.

Position	^1^H		^13^C	
	δ_H_ (multiplicity, *J* in Hz)		δ_C_	
	Experimental(CDCl_3_)	Reference [23](CDCl_3_)	Experimental(CDCl_3_)	Reference [23](CDCl_3_)
1	-	-	120.8	120.7
2	6.64 (*s*)	6.64 (*s*)	109.7	109.6
3	-	-	146.4	146.2
3a	5.88 (*s*)	5.88 (*s*)	101.0	100.9
4	-	-	141.0	140.9
5	6.51 (*s*)	6.52 (*s*)	95.0	94.8
6	-	-	152.2	152.0
1’	3.28 (*d*, 6.8)	3.28 (*d*, 6.6)	34.0	33.9
2’	5.87–5.97 (*m*)	5.89–5.97 (*m*)	137.3	137.2
3’	4.99–5.06 (*m*)	4.99–5.06 (*m*)	115.3	115.2
6-OMe	3.75 (s)	3.75 (s)	56.6	56.5

In the ^1^H-NMR spectrum (Table 1), the presence of two aromatic protons at δ_H_ 6.64 (1H, *s*, H-2) and δ_H_ 6.51 (1H, *s*, H-5) which appeared as singlets, suggested that the protons are at meta and para position. Two sets of multiplets were observed at δ_H_ 5.87–5.97 (1H, *m*) and δ_H_ 4.99–5.06 (2H, *m*) corresponding to vinyl protons, H-2’ and H_2_-3’ respectively. The presence of an upfield doublet at δ_H_ 3.28 (2H, *d*, *J* = 6.8 Hz, H_2_-1’) which correlated with the carbon signal at δ_C_ 34.0 (C-1’) in the HSQC spectrum coupled with the vinyl proton of H-2’ indicated the existence of an allyl group. In addition, a downfield singlet was observed at δ_H_ 5.88 (2H, *s*, H_2_-3a) thus suggesting the presence of a methylenedioxy group. A singlet representing the methoxyl protons attached to C-6 appeared at δ_H_ 3.75 (1H, *s*).

**Fig 5:**
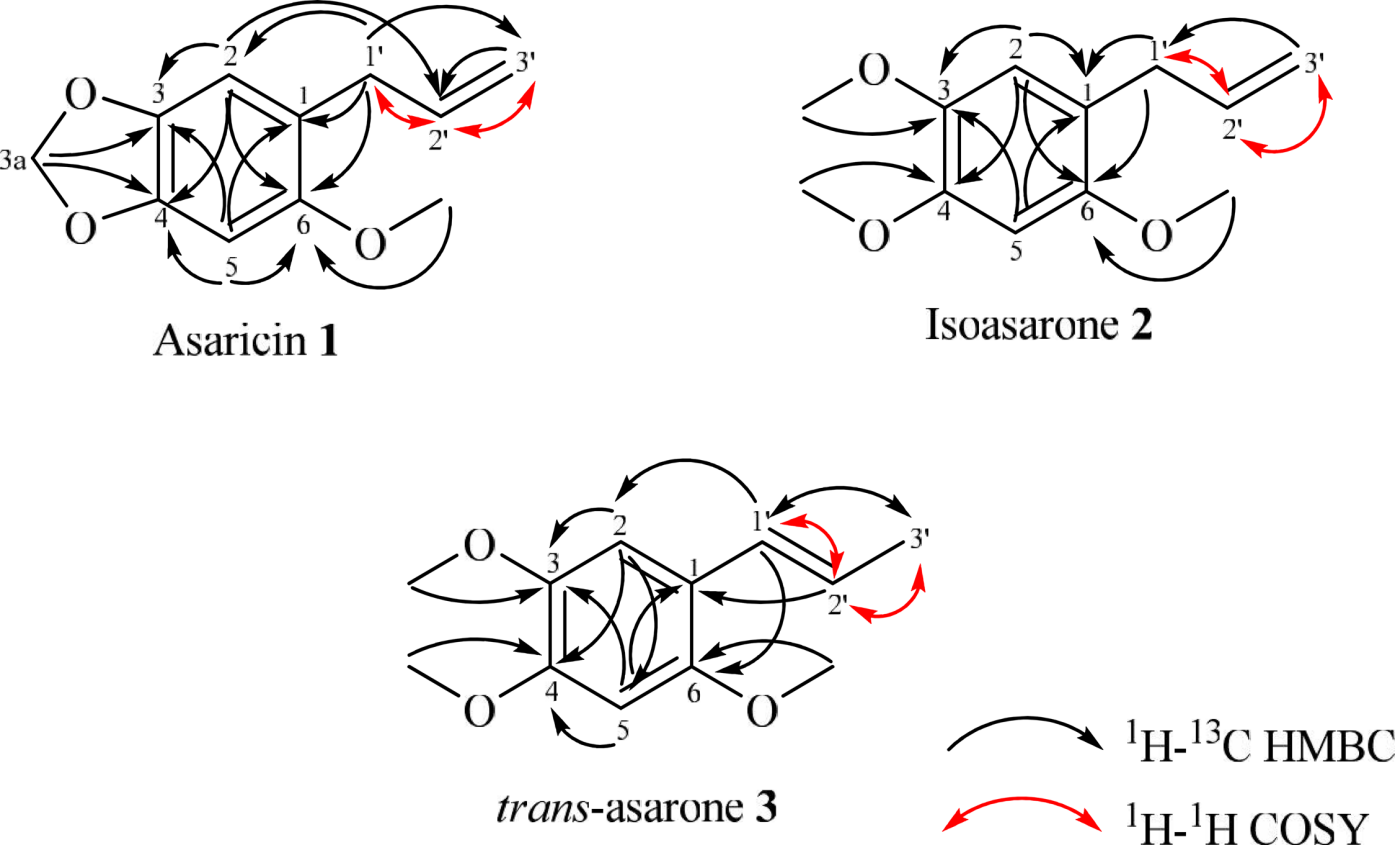
HMBC and COSY correlation of asaricin 1, isoasarone 2 and *trans*-asarone 3.

The ^13^C-NMR spectrum of asaricin **1** showed a total of eleven carbon signals; one methyl, three methines, three methylenes and four quaternary carbons. The methoxyl carbon resonated at δ_C_ 56.6 and the HMBC spectrum showed correlation of the methoxyl protons (OMe) with C-6 (δ_C_ 152.2), therefore indicated the connectivity of methoxyl group with the aromatic carbon, C-6 (Fig 5). Besides, the HMBC correlation of H_2_-3a with C-3 (δ_C_ 146.4) and C-4 (δ_C_ 101.1) inferred that the methylene group is attached to the quaternary carbons C-3 and C-4 of the benzene ring. The methylene protons of the allyl group is connected to the benzene ring at C-1 which can be deduced from the HMBC correlation between H_2_-1’ with C-1.

The spectroscopic data obtained (Table 1) were consistent with those found in the literature [23], thus confirming the identity of arsaricin **1** (Fig 4).

Isoasarone **2** was also isolated as an optically inactive yellow oil. Its molecular formula was confirmed as C_12_H_16_O_3_ from the LC-MS-IT-TOF which revealed a pseudomolecular ion peak [M+Na]^+^ at *m/z* 231.0258. Absorption bands at 1205 and 1038 cm^−1^ were observed in IR spectrum which were due to asymmetric and symmetric C-O-C stretching vibration [38].

In the ^1^H-NMR spectrum, the two aromatic protons, a methoxyl protons and an allyl group of isoasarone **2** exhibited the similar pattern with that of asaricin **1** except the absence of methylenedioxy group in isoasarone **2**. Instead two more singlets were observed at δ_H_ 3.84 and 3.89 corresponding to methoxyl protons which attached to C-3 and C-4 respectively as compared to arsaricin **1**.

The ^13^C-NMR spectrum of isoasarone **2** showed a total of twelve carbon signals; three methyl, three methines, two methylenes and four quaternary carbons. The three methoxyl protons which are correlated with C-3 (δ_C_ 56.8), C-4 (δ_C_ 56.4) and C-6 (δ_C_ 56.8) respectively are shown in HMBC spectrum (Fig 5).

From the analysis of spectroscopic data obtained (Table 2) and comparison with the literature values [24], the structure of isoasarone **2** (Fig 4) was confirmed without doubt.

**Table 2:** ^1^H-NMR (400 MHz) and ^13^C-NMR (100 MHz) spectral data of isoasarone 2 in CDCl_3_.

Position	^1^H		^13^C	
	δ_H_ (multiplicity, *J* in Hz)		δ_C_	
	Experimental(CDCl_3_)	Reference [24](CDCl_3_)	Experimental(CDCl_3_)	Reference [24](CDCl_3_)
1	-	-	120.2	120.3
2	6.70 (*s*)	6.70 (*s*)	114.1	114.3
3	-	-	143.2	143.3
4	-	-	148.1	148.2
5	6.54 (*s*)	6.54 (*s*)	98.2	98.4
6	-	-	151.5	151.6
1’	3.30 (*d*, 6.4)	3.30 (*dm*, 6.4)	33.8	33.8
2’	5.92–6.02 (*m*)	5.97 (*m*)	137.5	137.5
3’	5.02–5.07 (*m*)	5.04 (*m*)	115.4	115.4
3-OMe	3.84 (*s*)	3.83 (*s*)	56.8	56.8
4-OMe	3.89 (s)	3.88 (*s*)	56.4	56.5
6-OMe	3.81 (s)	3.81 (s)	56.8	56.8

*Trans*-asarone **3** was also isolated as an optically inactive yellow oil. The LCMS-IT-TOF mass spectrum displayed a pseudomolecular ion peak [M+H]^+^ at *m/z* 208.2625 which was in agreeable to the molecular formula of C_12_H_16_O_3_. The IR spectrum of *trans*-asarone **3** showed absorption peaks at 1202, 1033 and 970 cm^−1^ which indicated asymmetric and symmetric C-O-C and *trans-*double bond stretching, respectively [25, 38].

The ^1^H and ^13^C-NMR spectra of *trans*-asarone **3** showed very similar profile with that of isoasarone **2**. However, in ^1^H-NMR spectrum, a slight difference were observed in the signals of H-1’, H-2’ and H_3_-3’. In isoasarone **2**, one may observe the presence of three olefinic protons as compared to only two olefinic protons in *trans*-asarone **3**. In addition, the ^13^C-NMR spectrum of isoasarone **2** showed the presence of one sp^2^ methine and one sp^2^ methylene whereas in *trans*-asarone **3**, two sp^2^ methine were observed (δ_H_ 6.11, 1H, *dq*, *J* = 16.0, 6.9 Hz, H-2’ and δ_H_ 1.89, 3H, *dd*, *J* = 6.9, 1.8 Hz, H_3_-3’). These suggest that the terminal olefinic in isoasarone **2** is replaced with a 1’-propenyl group where the double bond is in the middle of the chain.

The ^13^C-NMR and DEPT spectra of *trans*-asarone **3** showed a total of twelve carbon signals; four methyl, four methines and four quaternary carbons. The COSY correlation between H_3_-3’ and H-2’ indicated the methyl group is attached to the *trans*-double bond of H-2’ (Fig 5). Complete ^1^H and ^13^C-NMR assignments (Table 3) were established by thorough analysis of COSY, HMBC and HSQC data.

**Table 3:** ^1^H-NMR (400 MHz) and ^13^C-NMR (100 MHz) spectral data of *trans*-asarone 3 in CDCl_3_.

Position	^1^H		^13^C	
	δ_H_ (multiplicity, *J* in Hz)		δ_C_	
	Experimental(CDCl_3_)	Reference [25](CDCl_3_)	Experimental(CDCl_3_)	Reference [25](CDCl_3_)
1	-	-	119.2	118.3
2	6.95 (*s*)	6.91 (*s*)	109.9	109.2
3	-	-	143.5	142.6
4	-	-	148.9	148.0
5	6.50 (*s*)	6.45 (*s*)	98.1	97.3
6			150.8	149.9
1’	6.66 (*dq*, 16.0, 1.8)	6.64 (*dq*, 16.0, 1.5)	125.2	124.4
2’	6.11 (*dq*, 16.0, 6.9)	6.02 (*dq*, 16.0, 6.2)	124.6	123.4
3’	1.89 (*dd*, 6.9, 1.8)	1.87 (*dd*, 6.2, 1.5)	19.0	18.7
3-OMe	3.86 (*s*)	3.81 (*s*)	56.9	56.1
4-OMe	3.89 (s)	3.84 (*s*)	56.3	55.1
6-OMe	3.83 (s)	3.77 (s)	56.7	55.7

From the analysis of the spectroscopic data obtained and comparison with the literature values [25], the identity of *trans*-asarone **3** was ensured.

### Larvicidal activity

**Fig 6:**
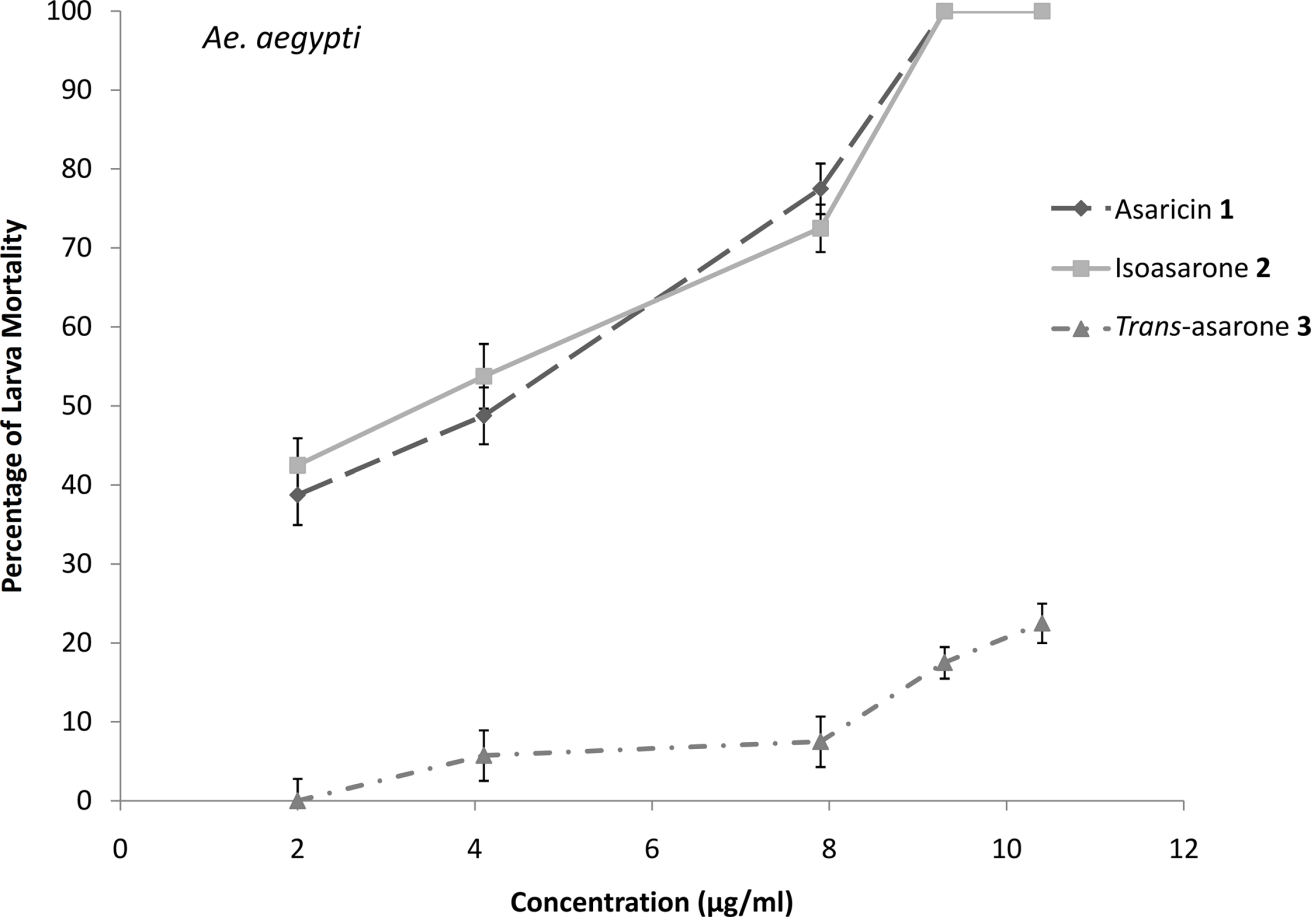
Percentage of larvicidal activity of three tested compounds in bioassay against *Ae*. *aegypti*. Each bar represents the mean ± standard error of four replicate. (p = 0.05).

**Fig 7:**
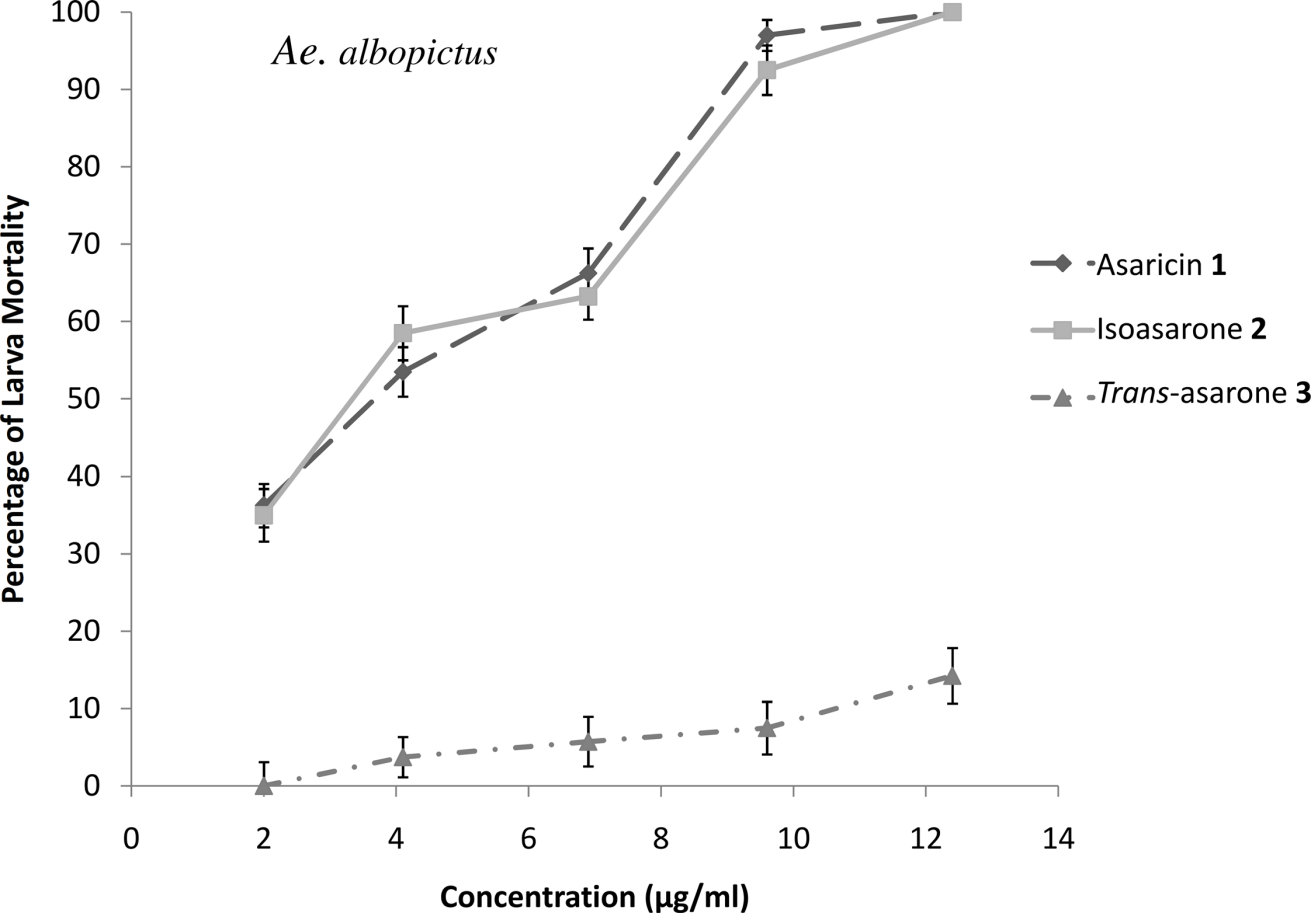
Percentage of larvicidal activity of three tested compounds in bioassay against *Ae*. *albopictus*. Each bar represents the mean ± standard error of four replicate. (p = 0.05).

**Fig 8:**
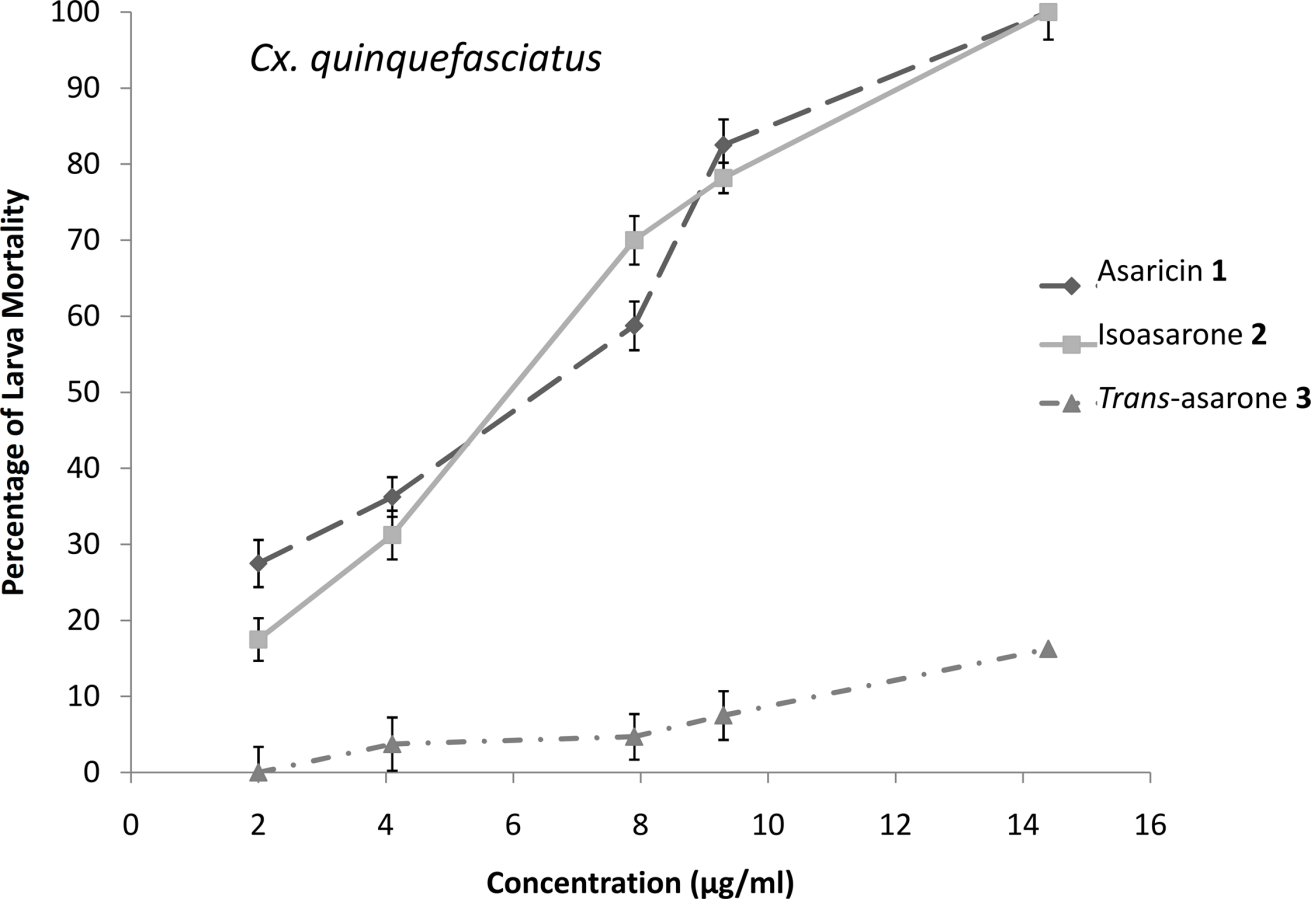
Percentage of larvicidal activity of three tested compounds in bioassay against *Cx*. *quinquefasciatus*. Each bar represents the mean ± standard error of four replicate. (p = 0.05).

The results of the larvicidal activity of the three phenylpropanoids against *Ae*. *aegypti*, *Ae*. *albopictus* and *Cx*. *quinquefasciatus* larvae are shown in Figs 6–8. The potency of the larvicidal activity of asaricin **1** and isoasarone **2** against *Ae*. *aegypti*, *Ae*. *albopictus* and *Cx*. *quinquefasciatus* larvae increases in a dose dependent manner. The larvicidal activity were also expressed as percentage of mortality, with dosage of ≤ 9.3 μg/mL for both compounds; asaricin **1** and isoasarone **2**, causing up to 100% and 90% mortality in *Ae*. *aegypti* and *Ae*. *albopictus* larvae respectively. Comparatively *Cx*. *quinquefasciatus* was the most resistant larvae in this assay; 9.3 μg/mL dosage could only cause up to 80% mortality. *Trans*-asarone **3** was less potent in all tested larvae whereby with 9.3 μg/mL, it only caused 5–20% mortality in all tested larvae (Figs 6–8).

### Ovicidal activity

**Fig 9:**
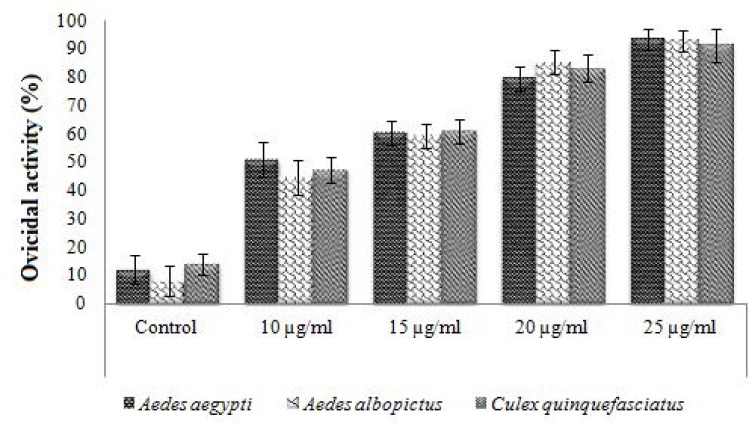
Ovicidal activity of asaricin 1 against *Ae*. *aegypti*, *Ae*. *albopictus* and *Cx*. *quinquefasciatus*.

**Fig 10:**
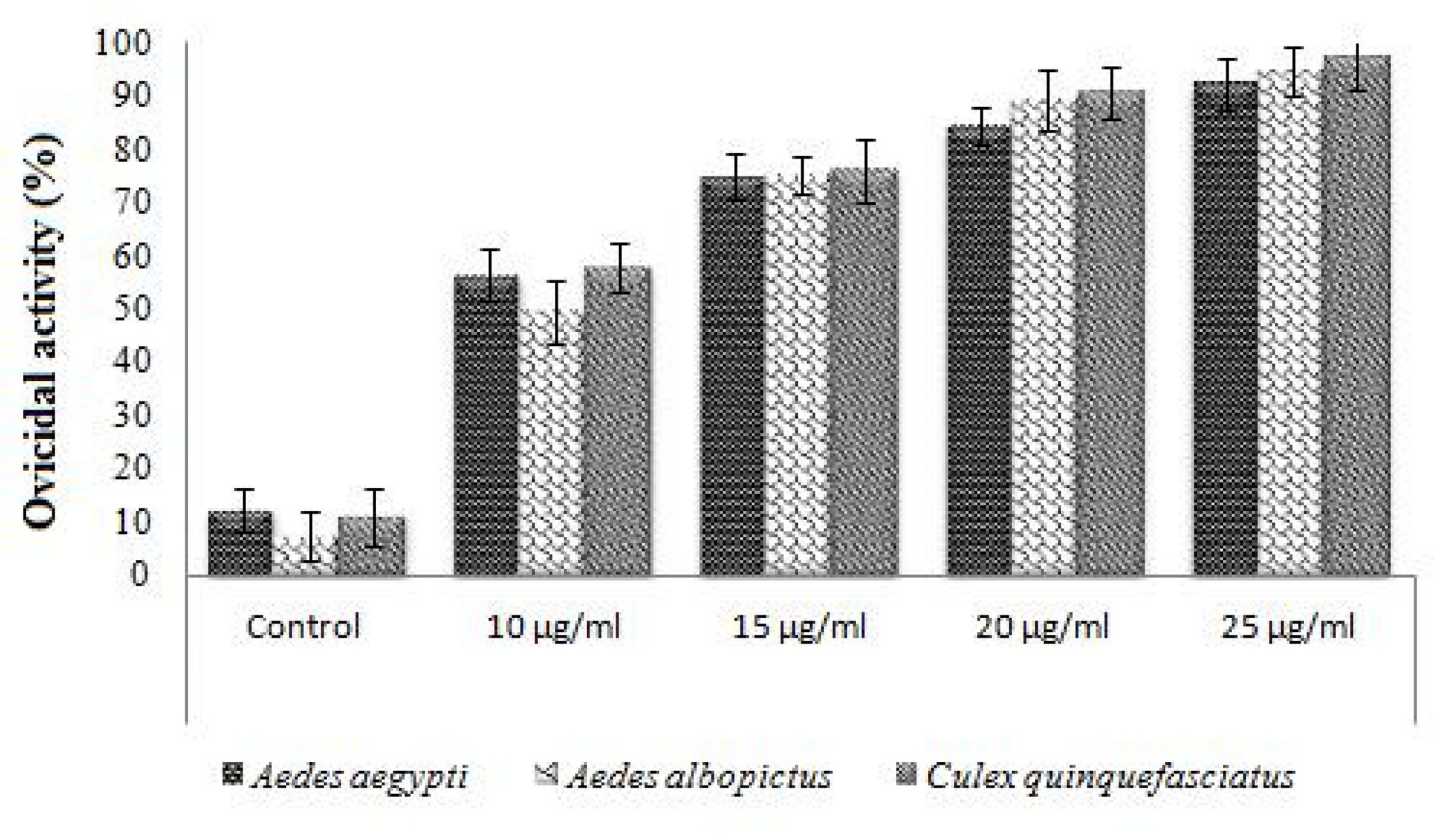
Ovicidal activity of isoasarone 2 against *Ae*. *Aegypti*, *Ae*. *albopictus* and *Cx*. *quinquefasciatus*.

Asaricin **1**, isoasarone **2** and *trans*-asarone **3** were studied for use as natural insecticide to inhibit the mosquito eggs from hatching. Statistical differences were observed (*p* < 0.05) among the efficacy averages of percentage for each compound. As shown in Figs 9 and 10, asaricin **1** and isoasarone **2** showed potent ovicidal activity up to 95% on *Ae*. *aegypti*, *Ae*. *albopictus* and *Cx*. *quinquefasciatus* at their highest dosage of 25 μg/mL. The ovicidal response of *Ae*. *aegypti*, *Ae*. *albopictus* and *Cx*. *quinquefasciatus* towards asaricin **1** at 25 μg/mL were similar by comparing the mean percentage of the ovicidal activity. The highest inhibition for asaricin **1** was observed at 25 μg/mL (Fig 9). Ovicidal effect of isoasarone **2** on *Ae*. *aegypti*, *Cx*. *quinquefasciatus* and *Ae*. *albopictus* was relatively strong. The inhibition range was 80 to 90% at 20 μg/mL and more than 90% at 25 μg/mL (Fig 10). Comparatively, ovicidal activity of asaricin **1** was slightly lower than isoasarone **2** at 15 μg/mL. *Trans*-asarone **3** had no effect on egg hatchability of the three mosquito species tested even at higher dosage of 100 μg/mL. Ovicidal test of asaricin **1** and isoasarone **2** within the range of 10 to 25 μg/mL showed potent inhibition on the development from egg to larvae.

### Repellent activity

**Fig 11:**
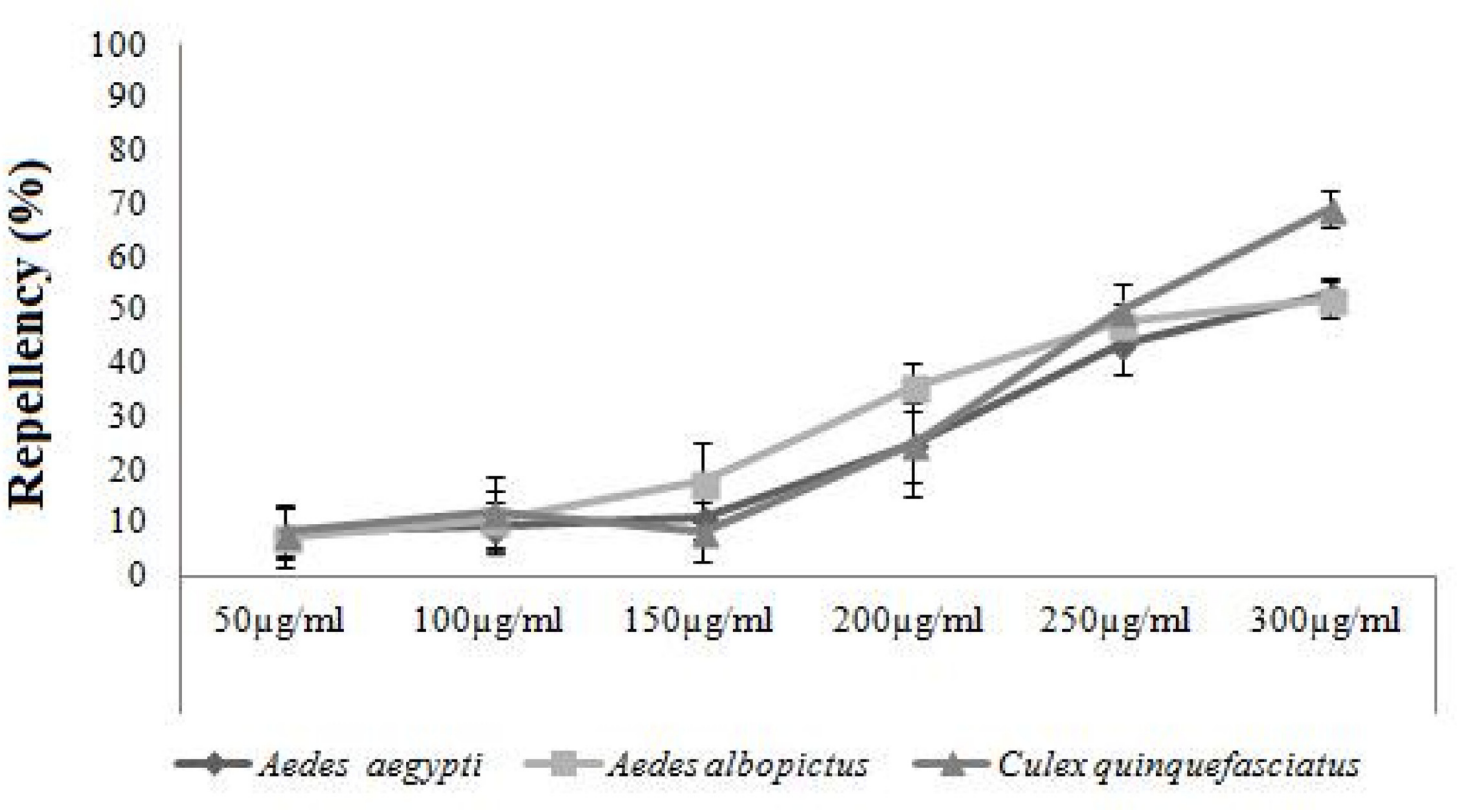
Repellent activity of asaricin 1 on *Ae*. *Aegypti*, *Ae*. *albopictus* and *Cx*. *quinquefasciatus*.

**Fig 12:**
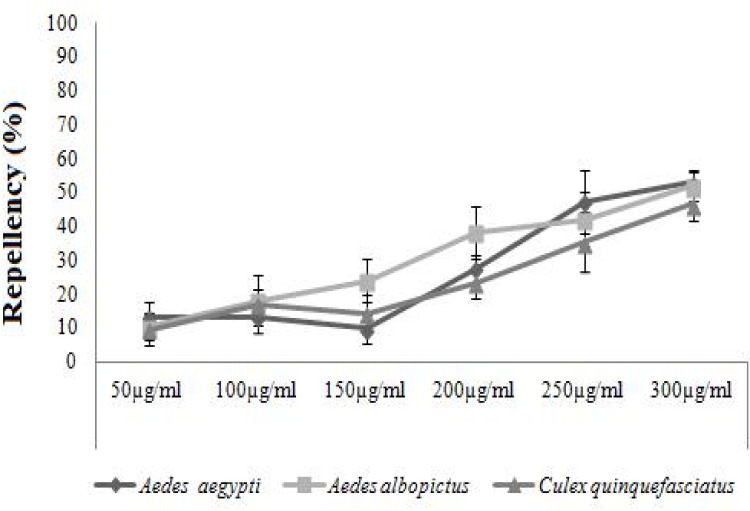
Repellent activity of isoasarone 2 on *Ae*. *Aegypti*, *Ae*. *albopictus* and *Cx*. *quinquefasciatus*.

Asaricin **1** was found to be very weak as a repellent agent. Even at high dosage of 300 μg/mL, its repellence activity was below 65%. As shown in Fig 11, the repellent activity was very low at the initial stage of exposure. Isoasarone **2** was slightly stronger than asaricin **1**; however, at high dosage of 300 μg/mL, the percentage of repellency was below 55% for *Ae*. *aegypti* and *Ae*. *albopictus*. *Cx*. *quinquefasciatus*showed more sensitivity in this test and it was more susceptible than *Ae*. *aegyptiAe*. *albopictus* (Fig 12). Repellent activity of *trans*-asarone **3** was not significant and they did not show any sign of repellent activity. Repellent activity of asaricin **1** and isoasarone **2** between the concentration ranges of 50 to 150 μg/mL was very weak. They showed some repellent activity only at concentration of 300 μg/mL which was considered very weak.

### IC_50_ value and acetylcholinesterase inhibition assay

**Fig 13:**
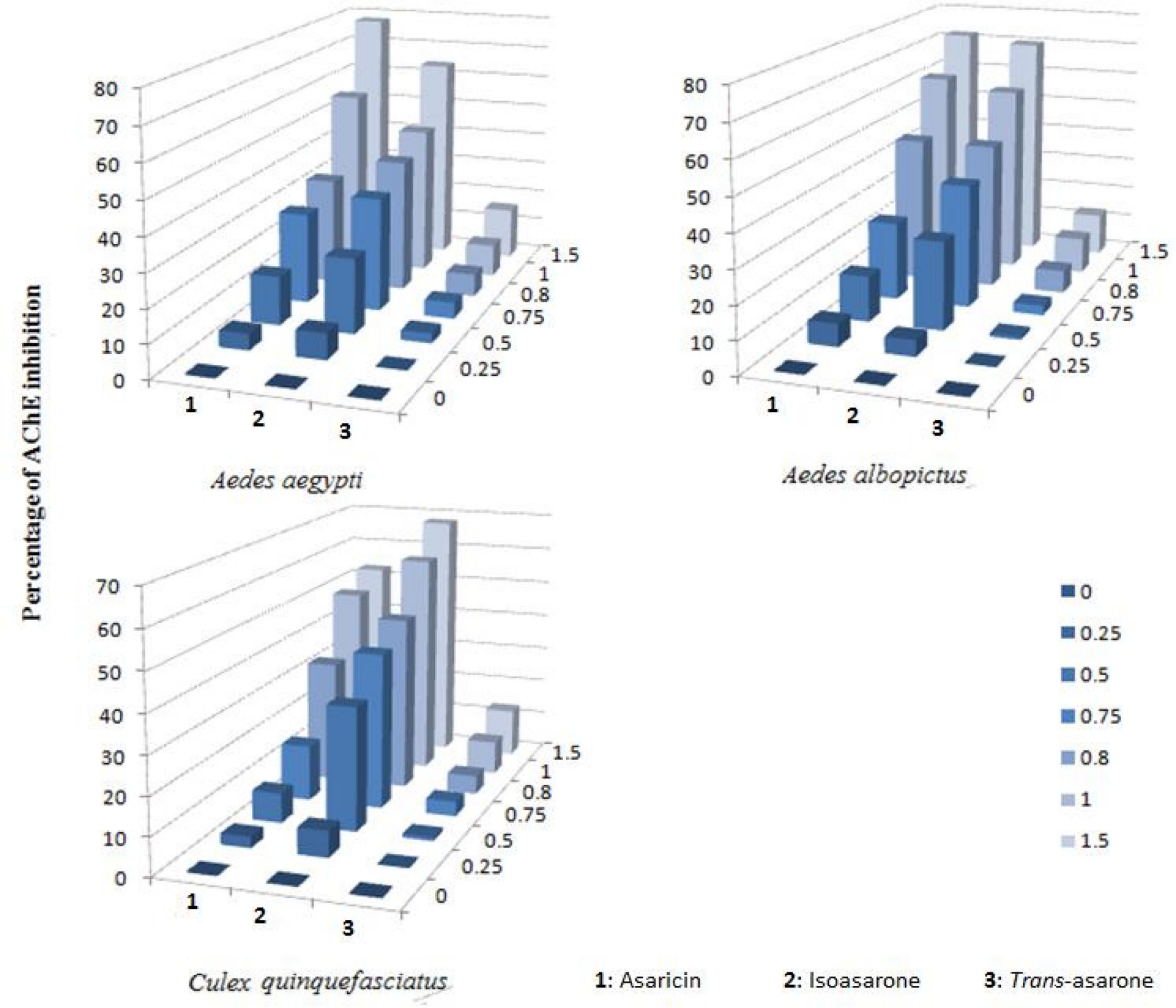
Inhibitory effect on acetylcholinesterase. Inhibition of acetylcholinesterase (AChE) extracted from *Ae*. *aegypti*, *Ae*. *albopictus* and *Cx*. *quinquefasciatus* larvae by three phenylpropanoid, was measured by acetylthiocholine chemical assay hydrolysis at 25°C. Each bar represents the mean of triplicate samples of three independent experiments (p = 0.05).

Asaricin **1,** isoasarone **2** and *trans*-asarone **3** were selected for AChE inhibitory activity in serial concentrations of 0.25–1.5 μg/mL. Absorbance values greater than 0.20 by monitoring at 412 nm according to the procedure described by Ellman represented the inhibition of acetylcholinesterase in *Ae*. *aegypti*, *Ae*. *albopictus* and *Cx*. *quinquefasciatus* [29]. Asaricin **1** and isoasarone **2** were highly active and inhibited AChE in a dose-dependent manner (Fig 13) and chi-square values calculated during the analysis did not show any heterogeneity of the response. The statistical analysis showed meaningful relation between the inhibition of AChE and the percentage of mortality. Asaricin **1** and isoasarone **2** AChE inhibition and their level of toxicity was significant and negatively correlated (*p* > 0.05). The IC_50_ of the tested compounds were given in Table 4. Asaricin **1** exhibited the highest inhibition against AChE extracted from *Ae*. *aegypti*, *Ae*. *albopictus* and *Cx*. *quinquefasciatus* with IC_50_ of 0.73–2.24 μg/mL followed by isoasarone **2** which relatively showed strong inhibition of AChE while *trans*-asarone **3** had the lowest inhibition capability (Table 4). Therefore, the AChE inhibition effect of inhibitors can also be characterized by the slope of the log-probit curve. The slope of the enzyme inhibition curves is showing relative response to treatment within the insects enzyme tested.

**Table 4:** Acetylcholinesterase inhibitory activities of asaricin 1, isoasarone 2 and *trans*-asarone 3 from *Piper sarmentosum*.

Compounds	AChE enzyme inhibition								
	*Aedes aegypti*			*Aedes albopictus*			*Culex quinquefasciatus*		
	IC_50_ (μg/mL)	χ2(df)	Slope ± S.E	IC_50_ (μg/mL)	χ2(df)	Slope ± S.E	IC_50_ (μg/mL)	χ2(df)	Slope ± S.E
Asaricin **1**	0.73( 0.42 to 0.93)	2.24	2.07±0.13	0.84(0.61 to 1.05)	2.55	2.25±0.14	2.24(1.83 to 2.5)	1.93	2.50±0.14
Isoasarone **2**	0.92( 0.73 to 1.22)	2.92	2.41±0.15	1.65(1.12 to 2.1)	3.90	2.57±0.14	1.87(1.56 to 2.4)	2.67	2.29±0.13
*Trans*-asarone **3**	15.75(4.6 to24.3)	7.51	3.63±0.14	9.75(7.5 to 13.1)	4.22	2.00±0.16	17.4(12.6 to 24.4)	5.83	2.46±0.13

Note: Values are mean ± SE of four times tests. For example 0.73 (0.42 to 0.93) means that IC_50_ value of asaricin **1** was 0.73 μg/mL and (0.42 to 0.93) is 95% confidence interval of this calculation. IC_50_ values significantly different (*p* ˃ 0.05) are based on non-overlap of the 95% CL. probit analysis at *p* > 0.05 using the program POLO-Plus 2.0 (LeOra-Software 2005).

### Computational investigation for phenylpropanoids compounds toward the three binding sites

**Fig 14:**
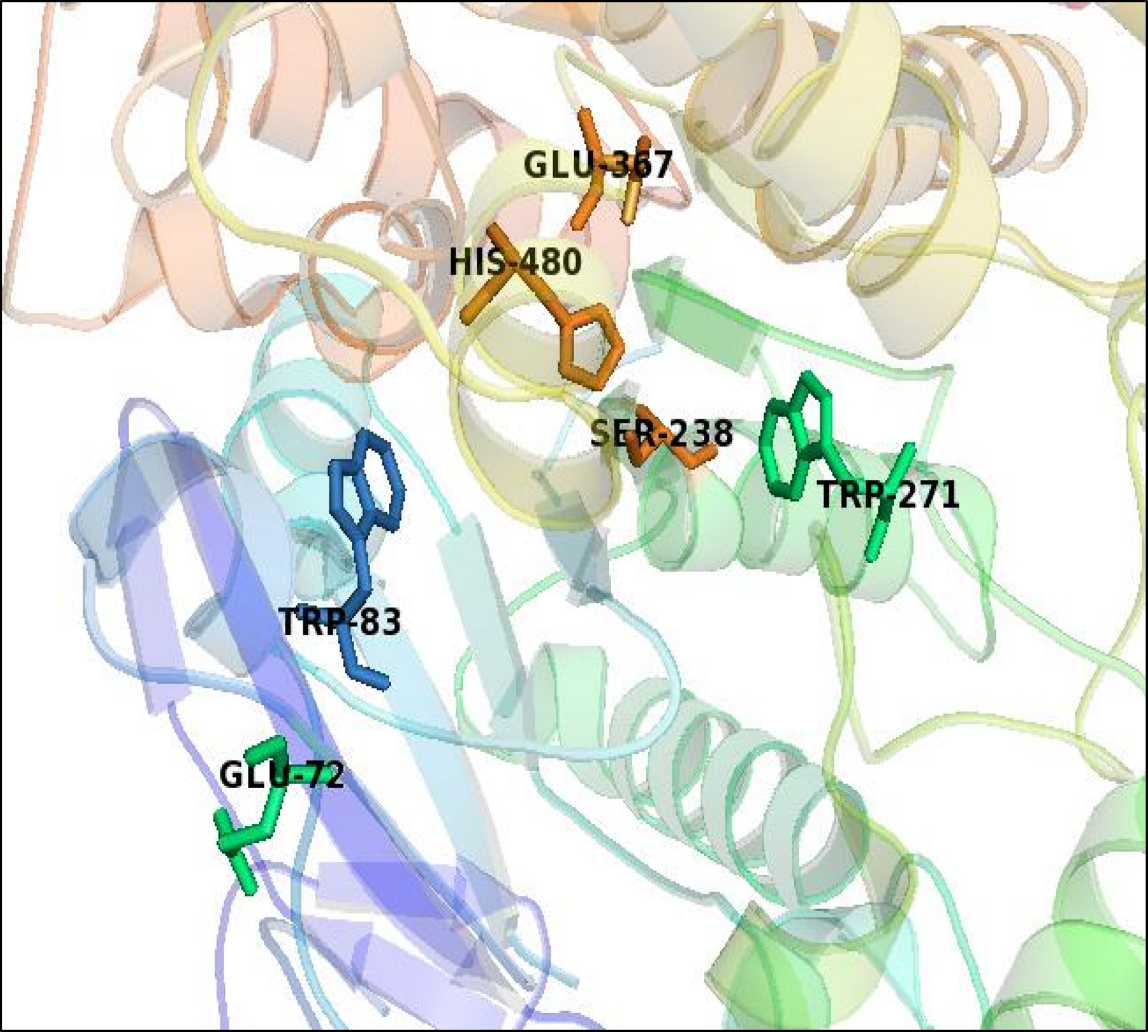
Binding residues of three binding sites of AChE enzyme, blue, orange and lime green residues depict the anionic binding site (TRP83), catalytic binding site (SER238, GLU367, HIS480) and peripheral binding site (TRP271) respectively.

**Fig 15:**
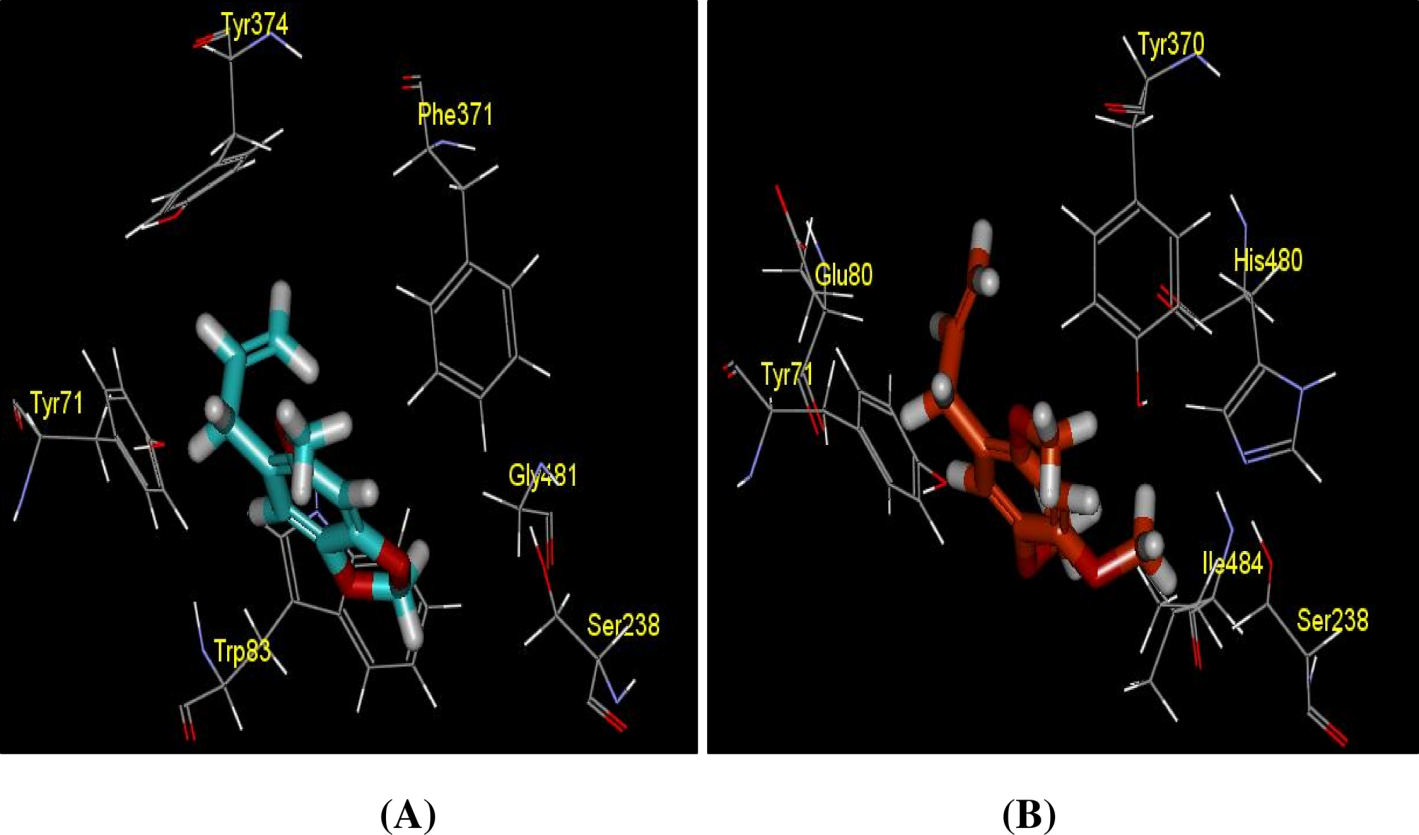
Docking structures of compound (A) asaricin **1** and (B) isoasarone **2** toward Catalytic site of AChE and their closed contact residue interaction. Residue interacted with compound at < -2kcal/mol were shown only.

**Fig 16:**
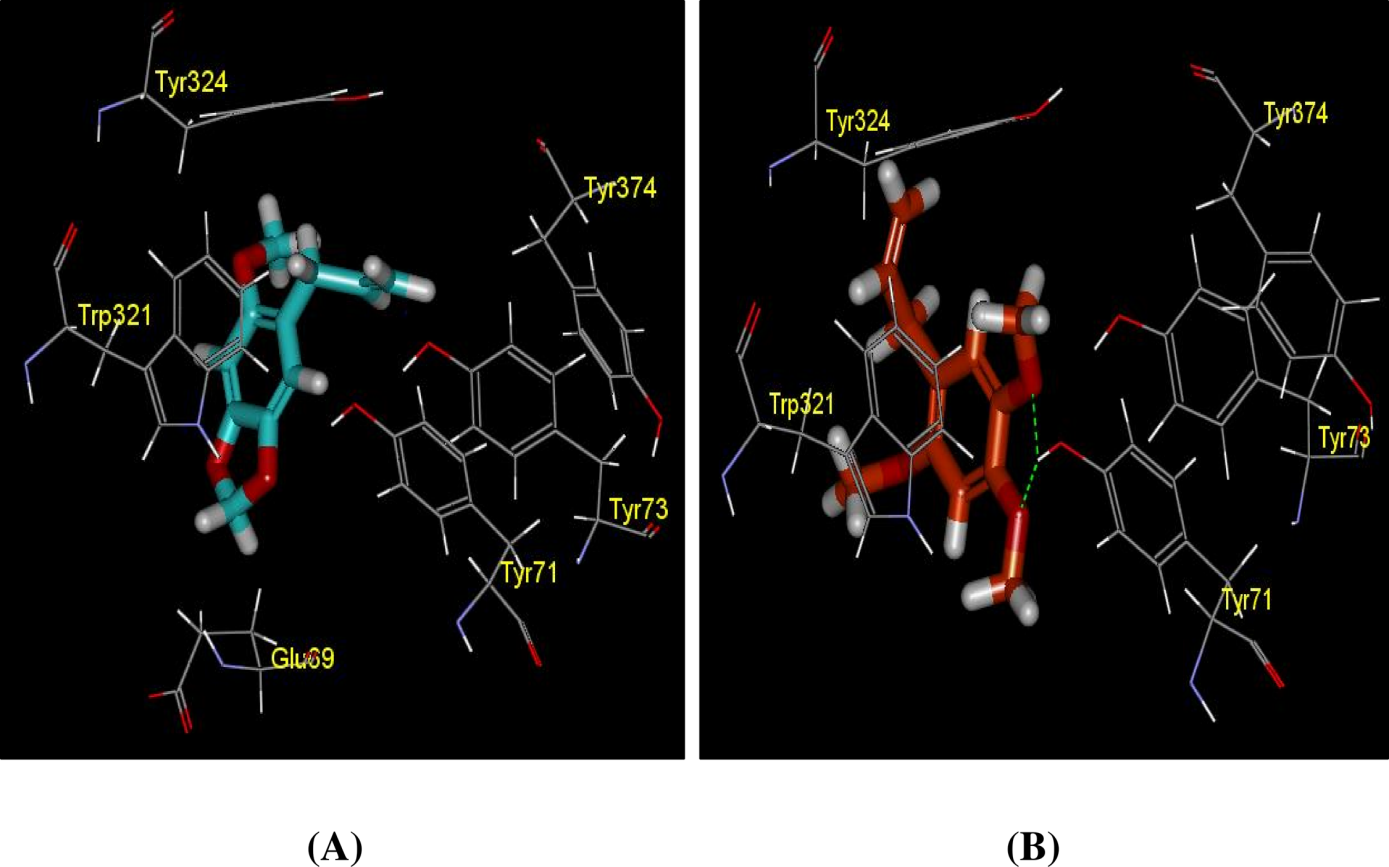
Docking structures of compound (A) asaricin **1** and (B) isoasarone **2** toward peripheral site of AChE and their closed contact residue interaction with the hydrogen bond interactions at TRP77:HH: ACO14 (2.17Å) and TRP77:HH: ACO12 (2.27Å) in (b) Residue interacted with compound at < -2 kcal/mol were shown only.

**Fig 17:**
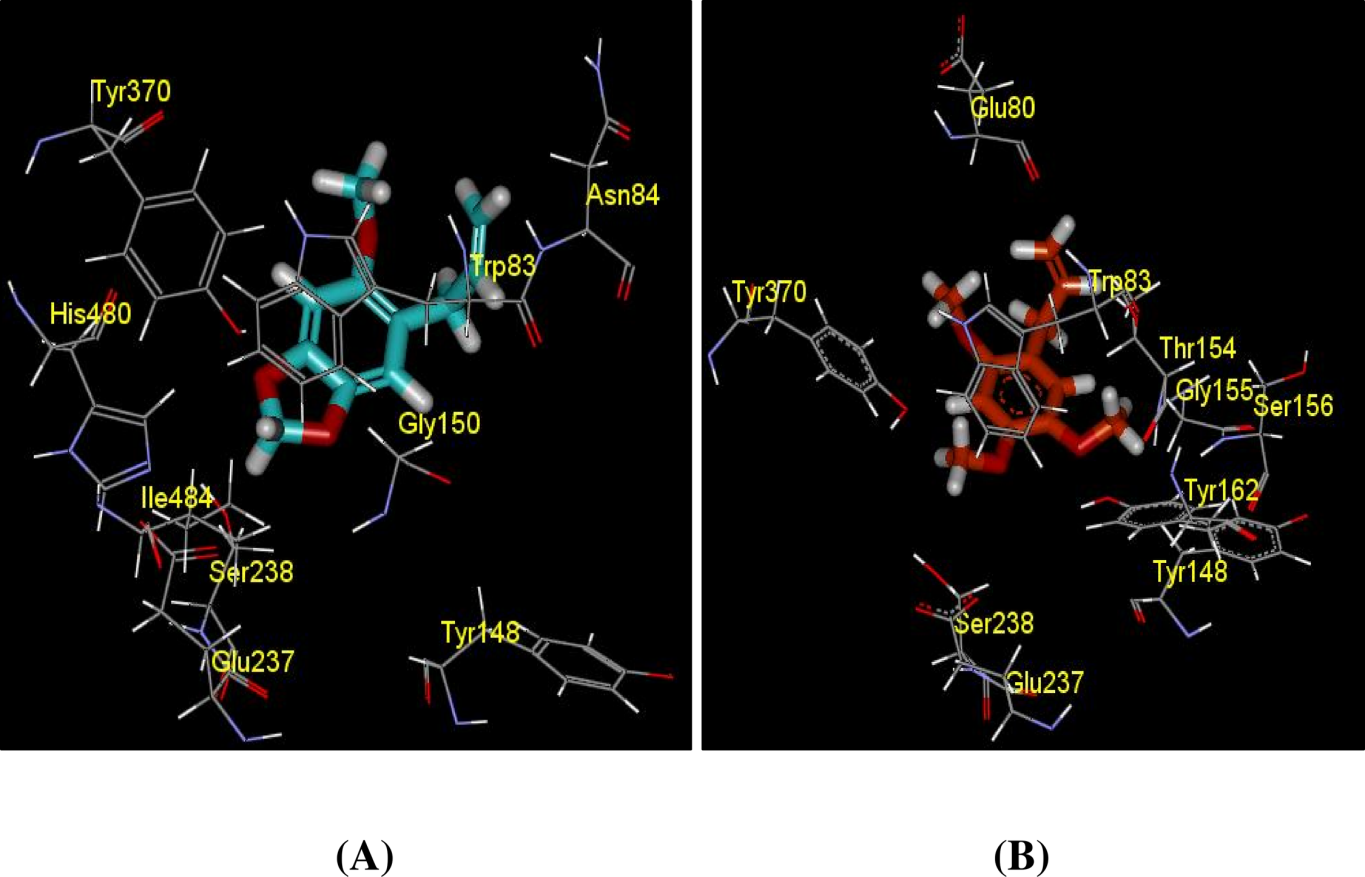
Docking structures of compound (A) asaricin **1** and (B) isoasarone **2** toward Anionic site of AChE and their closed contact residue interaction. Residue interacted with compound at < -2 kcal/mol were shown only.

Binding energy evaluation provided a correlation to the activity performed at the experimental stage. The best docked pose with the lowest binding energy was selected from series of poses generated after calculating their binding energy. The binding energy follows the equation as Binding Energy = Energy of Complex—Energy of Ligand—Energy of Receptor. The more negative the binding energy, the better the binding activity. In another word, the binding between the compound and the target will be more favourable. Binding affinity calculated from Autodock/vina revealed that asaricin **1 **and isoasarone **2 **bound in the anionic site stronger with -6.7 kcal/mol and -6.0 kcal/mol respectively as compared to CAS (asaricin **1**: -4.7, isoasarone **2**: -3.9 kcal/mol) and PAS (asaricin **1**: -5.4, isoasarone **2**: -5.2 kcal/mol) site (Table 5). Apart from the interaction energy of the enzyme and compounds at the active sites, interaction energy for the residues within 3 Å region was also calculated as a more precise indication on the binding strength of asaricin **1** and isoasarone **2**. Summation of the interaction energy of each residue with asaricin **1** and isoasarone **2** was compared in Tables 6–8. 3Å IE of asaricin **1** and isoasarone **2** in anionic site is -31.43 kcal/mol and -36.49 kcal/mol, respectively which is stronger than CAS (asaricin **1**: -14.26, isoasarone **2**: -16.51 kcal/mol) and PAS (asaricin **1**: -20.43, isoasarone **2**: -14.52 kcal/mol). Three binding sites of the enzyme AChE were illustrated in Fig 14, while Figs 15–17 facilitates visualization of the molecular interaction of compounds bounded in all the three binding pockets. Common residues in binding for both compounds with interaction energy lower than—2 kcal/mol are TYR 71, TRP 83 for CAS site, TYR71, TYR 73, TRP 321, TYR 324 and TYR 374 for PAS site, and TRP 83, TYR 148, GLU 237, SER 238 and TYR 370 for anionic site.

**Table 5:** Binding interaction energy (kcal/mol) of compounds from Autodock Vina toward different binding sites and their related experimental activities for AChE for *Ae*. *aegypti*, *Ae*. *albopictus* and *Cx*. *quinquefasciatus*, respectively.

Inhibitors	CAS site	PAS site	Anionic binding site (W83)	AChE IC_50_ for *Ae. Aegypti/Ae. Albopictus/Cx. quinquefasciatus* (µg/mL)
Asaricin **1**	-4.7	-5.4	-6.7	0.73/0.84/2.20
Isoasarone **2**	-3.9	-5.2	-6.0	0.92/1.65/1.87
(Double bond is at the end of R-group)				

**Table 6:** The interaction energy of phenolic compounds toward AChE catalytic site.

Residue	IE	VDW	Electrostatics	Residue	IE	VDW	Electrostatics
	(kcal/mol)	(kcal/mol)	(kcal/mol)		(kcal/mol)	(kcal/mol)	(kcal/mol)
**AChE**							
**CAS site**							
**1**	** **	** **	** **	**2**	** **	** **	** **
ARG 70	2.02	-0.083	2.1	ARG 70	-1	-0.073	-0.93
**TYR 71**	-6.27	-3.34	-2.93	**TYR 71**	-4.23	-3.39	-0.88
**GLU 80**	3.29	-1.52	4.81	**GLU 80**	-1.41	-1.86	0.45
**TRP 83**	-3.08	-2.72	-0.37	**TRP 83**	-2	-1.86	-0.14
GLY 149	-1.2	-0.84	-0.36	**GLY 149**	-1.39	-1.64	0.24
**GLY 150**	-0.34	-1.16	0.82	**GLY 150**	2.98	0.51	2.47
**GLY 151**	0.14	-0.85	0.99	**GLY 151**	-0.11	-0.82	0.71
THR 154	-0.85	-0.46	-0.38	THR 154	-0.35	-0.54	0.19
TYR 162	2.22	-0.38	2.6	TYR 162	1.66	-0.47	2.14
GLU 237	-1.32	-0.32	-1	GLU 237	-0.0037	-0.55	0.55
SER 238	-2.81	-0.38	-2.43	**SER 238**	-2.81	-0.82	-1.99
**TYR 370**	-1.46	-1.97	0.51	**TYR 370**	-5.04	-2.18	-2.86
**PHE 371**	-2.45	-1	-1.45	**PHE 371**	-0.81	-1.49	0.69
**TYR 374**	-2.85	-1.4	-1.45	**TYR 374**	2.08	-1.5	3.58
**TRP 472**	-1.24	-0.87	-0.37	**TRP 472**	-1	-0.95	-0.045
HIS 480	-1.05	-0.67	-0.38	**HIS 480**	-2.77	-1.25	-1.52
GLY 481	-2.05	-0.35	-1.7	GLY 481	-1.57	-0.4	-1.17
ILE 484	-1.32	-0.2	-1.13	ILE 484	-2.47	-0.31	-2.16
**3Å IE**	**-14.26**	**-14.83**	**0.56**	**3Å IE**	**-16.51**	**-17.25**	**0.71**
**Total IE**	**-83.21**	**-21.27**	**-61.93**	**Total IE**	**-100.29**	**-22.81**	**-77.48**

Note: The bold amino acid residues of AChE are involved in 3Å vicinity of compound upon binding.

**Table 7:** The interaction energy of phenolic compounds toward AChE peripheral site.

Residue	IE	VDW	Electrostatics	Residue	IE	VDW	Electrostatics
	(kcal/mol)	(kcal/mol)	(kcal/mol)		(kcal/mol)	(kcal/mol)	(kcal/mol)
AChE							
PAS							
**1**				**2**			
**GLU 69**	-8.36	-1.2	-7.16	**GLU 69**	5.06	-1.41	6.47
ARG 70	0.066	-0.28	0.35	ARG 70	-0.89	-0.89	0.0027
**TYR 71**	-2.22	-1.53	-0.69	**TYR 71**	-9.35	-2.98	-6.37
**TYR 73**	-3.76	-0.96	-2.79	TYR 73	-5.42	-1.46	-3.96
VAL 318	1.01	-0.71	1.72	**VAL 318**	0.082	-1.37	1.45
**TRP 321**	-4.04	-5.4	1.36	**TRP 321**	-7.42	-7.85	0.43
TYR 324	-2.85	-1.99	-0.86	**TYR 324**	-2.89	-2.045	-0.85
**TYR 374**	-2.05	-0.59	-1.46	TYR 374	-3.36	-0.23	-3.13
3Å IE	-20.43	-9.68	-10.74	3Å IE	-14.52	-15.66	1.13
Total IE	-66.33	-14.82	-51.51	Total IE	-78.41	-21.16	-57.25

Note: The bold amino acid residues of AChE are involved in 3Å vicinity of compound upon binding.

**Table 8:** The interaction energy of phenolic compounds toward AChE anionic site.

Residue	IE	VDW	Electrostatics	Residue	IE	VDW	Electrostatics
	(kcal/mol)	(kcal/mol)	(kcal/mol)		(kcal/mol)	(kcal/mol)	(kcal/mol)
**AChE**							
**Anionic site**							
**1**	** **	** **	** **	**2**	** **	** **	** **
**TYR 71**	-0.79	-2.41	1.62	**TYR 71**	0.41	-0.78	1.19
**GLU 80**	-0.69	-1.75	1.06	**GLU 80**	-6.62	-1.43	-5.19
**TRP 83**	-5.3	-5.034	-0.27	**TRP 83**	-6.31	-6.82	0.51
**ASN 84**	-2.51	-1.53	-0.98	**ASN 84**	-1.96	-2.35	0.39
TYR 148	-4.18	-0.2	-3.99	**TYR 148**	-3.66	-0.83	-2.83
**GLY 149**	-1.8	-1.21	-0.59	**GLY 149**	-0.075	-1.51	1.43
**GLY 150**	-2.64	-1.87	-0.76	**GLY 150**	-1.93	-2.3	0.37
GLY 151	0.68	-0.24	0.91	GLY 151	0.52	-0.23	0.75
**THR 154**	-0.99	-1.42	0.43	**THR 154**	-2.8	-2.27	-0.53
GLY 155	-0.24	-0.36	0.12	**GLY 155**	-2.42	-0.98	-1.44
SER 156	-1.25	-0.096	-1.16	**SER 156**	-2.86	-0.63	-2.23
TYR 162	-0.9	-0.51	-0.38	**TYR 162**	-4.93	-1.48	-3.45
**GLU 237**	-6.71	-0.83	-5.88	GLU 237	-2.15	-0.45	-1.7
**SER 238**	-3.18	-0.85	-2.33	SER 238	-2.24	-0.35	-1.88
**TYR 370**	-2.13	-1.98	-0.15	**TYR 370**	-3.33	-1.48	-1.84
PHE 371	-1.26	-0.36	-0.9	PHE 371	0.36	-0.31	0.67
TRP 472	1.78	-0.22	2	TRP 472	-1.84	-0.31	-1.53
**HIS 480**	-2.19	-1.24	-0.95	HIS 480	1.13	-0.65	1.78
**ILE 484**	-2.5	-0.48	-2.03	ILE 484	-0.53	-0.37	-0.16
**3Å IE**	**-31.43**	**-20.60**	**-10.83**	**3Å IE**	**-36.49**	**-22.86**	**-13.62**
**Total IE**	**-80.74**	**-26.569**	**-54.18**	**Total IE**	**-118.32**	**-29.94**	**-88.38**

Note: The bold amino acid residues of AChE are involved in 3Å vicinity of compound upon binding.

## Discussion

### The percentage of mortality, ovicidal, repellent and acetylcolinesterase activities

Extracts of many medicinal plants are known to have toxic effects on different species of vectors such as mosquitoes for example *Aegle marmelos* (Linn.), *Limonia acidissima* (Linn.), *Sphaeranthus indicus* (Linn.), *Sphaeranthus amaranthoides* (burm.f) [39]. The use of local medicinal plant extracts for mosquito control will reduce dependence on expensive imported products and maximize the local efforts to enhance public health [40]. The larvicidal tests were conducted against *Ae*. *aegypti*, *Ae*. *albopictus* and *Cx*. *quinquefasciatus* and results indicated the high potency of asaricin **1**, isoasarone **2** and mild activity of *trans*-asarone **3**. *In vitro* bioassay against *Ae*. *aegypti*, *Ae*. *albopictus* and *Cx*. *quinquefasciatus* larvae proved that the dosage that can cause 50% mortality was ≤ 7 μg/mL and 100% mortality was observed at concentration of ≤ 15 μg/mL which were relatively low for asaricin **1** and isoasarone **2** (Figs 6–8). *Cx*. *quinquefasciatus* larvae showed the highest resistance among the three tested species. Toxic action of these compounds could be the result of unspecified toxicity related to hydrophobicity and the generation of organic radicals and reactive oxygen species [41]. In previous studies on Piper family including *P*. *sarmentosum*, ovicidal activity of extracts and essential oils has been reported [42]. Based on literature review, this is the first report of highly effective larvicidal and ovicidal activity of specific phenylpropanoids compounds from *P*. *sarmentosum*. Asaricin **1** and isoasarone **2** showed reliable ovicidal activity against three mosquito species; *Ae*. *aegypti*, *Ae*. *albopictus* and *Cx*. *quinquefasciatus*. It is possible that this activity be significantly improved by future studies on formulation of the above compounds. On the other hand, the repellent activity was not as significant, this may be due to the structure of these compounds (hydrophilic head and hydrophobic tail). They may be useful in combining with other known natural repellent.

To clarify the mechanism of action of asaricin **1** and isoasarone **2**, AChE enzyme inhibition activity were evaluated and apparently asaricin **1** and isoasarone **2** showed the highest AChE inhibition. This result indicated that there is correlation between IC_50_ value and AChE inhibition value in *Ae*. *aegypti*, *Ae*. *albopictus* and *Cx*. *quinquefasciatus* larval stage; the lower the IC_50_ value represents the higher the AChE inhibition.

The significant negative correlation of asaricin **1** and isoasarone **2** toxicity with AChE inhibition suggest that asaricin **1** and isoasarone **2** may be considered as neuron toxic compounds. To the author’s knowledge, this is the first report on the larvicidal and AChE inhibition activity of asaricin **1,** isoasarone **2** and *trans*-asarone **3** against *Ae*. *aegypti*, *Ae*. *albopictus* and *Cx*. *quinquefasciatus*.

### Binding affinity and mode of interaction of compounds towards AChE

Binding energy and interaction energy computed from Autodock/vina and Discovery Studio programme, respectively would reveal the binding affinity and interaction mediated by the compounds within the binding sites of enzyme. The detailed interaction formed between the binding residues of enzyme towards the compounds could be dissected by calculating 3 Å interaction energy. Calculated interaction energy of both asaricin **1** and isoasarone **2** exhibited a negative value and also implying the binding affinity of the compounds to anionic site is higher compared to CAS site and PAS site, as summarized in Tables 6–8. The calculated binding energies were well correlated with experimental data that both compounds are active against AChE. Therefore, further analysis on the intermolecular interactions would explain the differences in binding activity in different binding site as showed in Figs 15–17.

#### Molecular interaction of asaricin 1 and isoasarone 2 toward AChE at the catalytic binding site

Binding affinity of asaricin **1** and isoasarone **2** in catalytic binding site obtained from from Autodock Vina is generally low, -4.7 kcal/mol and -3.9 kcal/mol, respectively (Table 5). It is also consistent with the high 3 Å interaction energy (IE) of asaricin **1** and isoasarone **2** at -14.26 and -16.51 kcal/mol as tabulated in Table 6. The intermolecular interactions mostly contributed by aromatic-aromatic ring interactions [43] as the compounds containing aromatic rings that favoured the ring-ring interaction. Asaricin **1** and isoasarone **2** were found interacting with TYR 71, TRO 83 and TYR 370 in similar manner. Stacking effect between the ring of the compounds and the ring of TYR 71 gave rise to very low interaction energy. It is however, distance between the rings gave energy contribution at different magnitude.

#### Molecular interaction of asaricin 1 and isoasarone 2 toward AChE at the PAS binding site

The binding affinity (Table 5) and 3 Å IE of the asaricin **1** and isoasarone **2** in peripheral binding sites (PAS) is slightly higher than the catalytic site (Table 7). Although asaricin **1** did not form hydrogen bond with any of the binding residues, however due to its elongated structure, it penetrates deeper into the binding site and making contact with GLU 69 with a relatively low energy. Whereas isoasarone **2** formed two hydrogen bonds with residue TYR 71 through atom O12 and O14 at distance of 2.17Å and 2.27Å, respectively and therefore, a very low interaction energy was observed, about -9.35 kcal/mol. Apart from that, the -OH group of residue TYR 73 directed itself towards the aromatic ring of the asaricin **1** and isoasarone **2** as presented in Fig 16. The presence of electron-donating group -OCH_3_ attached to the ring resulted in the electron density in the ring to be high and hence, created a negatively charged site that possibly come in contact with the hydrogen of the OH group of TYR 73 which was partially electron deficient. Distance between the ring of asaricin **1** and isoasarone **2** and hydrogen of TYR 73 gave rise to the difference in energy contribution. A great difference in opposition charge and short distance between the two interface groups would produce a tight binding. Another important binding residue was observed to be TRP 321. The ring structure of the two compounds interfaced with the rings of TRP 321 and therefore, initiated aromatic-aromatic interactions. Besides, isoasarone **2** was observed making a hydrogen contact with TRP 321 and it strengthens the interaction to a greater extend.

#### Molecular interaction of asaricin 1 and isoasarone 2 toward AChE at the anionic site

The calculated binding affinity and 3 Å IE for asaricin **1** and isoasarone **2** when they are accommodated in anionic site are very much different from other binding sites. Both of the compounds are having the highest binding affinity, lower than 6 kcal/mol (Table 5) in this binding pocket despite the absence of hydrogen bond formation. TRP 83 is highlighted at the first place due to its distinctive low energy as tabulated in Table 8. The ring of compounds and the ring of TRP 83 have just come into right position that encouraged the stacking as displayed in Fig 17. Ring-ring interactions also occurred between the compounds and TYR 370. The side chain of isoasarone **2** that containing a double bond, together with its adjacent side chain were observed to form a very large interactive surface to interact with the residues of the enzyme, THR 154, GLY 155, SER 156, TYR 162 within the binding site. It is therefore contributed to a lower 3 Å IE as compared to asaricin **1**. On the other hand, the elongated structure of asaricin **1** enabled it to be embedded deeper into the binding pocket to interact with GLU 237, SER 238 and ILE 484 with a much lower energy. Within the 3 Å binding vicinity, van der Waals interactions are more vital than electrostatics forces to accommodate the ligand in the binding site. The two compounds are found to be most active in this binding site most probably due to the ability in mediating interactions with more residues within 3 Å using a lower energy.

## Conclusion

Mosquito vector population control is a vast and daunting problem faced by populations all over the world especially those in tropical countries. Current study on *P*. *sarmentosum* bioactive compounds revealed the potential to enhance current control measures, and possibly contribute towards long term control of mosquito populations. Asaricin **1** and isoasarone **2** were the key derivatives that showed strong larvicidal and ovicidal activity in this study, against *Ae*. *aegypti*, *Ae*. *albopictus* and *Cx*. *quinquefasciatus* larvae. The AChE assay exhibited that asaricin **1** and isoasarone **2** have strong inhibition activity and docking studies brought more evidence and explanation for the possible mechanism of action and the insects response. The binding affinity of the compounds towards the three binding pockets of AChE were explored and compared. Binding affinity from Auto-dock/vina suggested that all three binding sites could be the possible targets for the compounds to interact. This has been further supported by calculated surface interaction energy within 3 Å region from the compound. The low binding interaction energy implied that asaricin **1** and isoasarone **2** were actually bounded strongly at all sites with the anionic site being the strongest. These findings are consistent with the laboratory results obtained on the AChE assay. In particular, the binding affinity at the anionic binding site revealed that asaricin **1** bind slightly stronger than isoasarone **2** which correlated to the IC_50_ value against AChE observed; IC_50_ for asaricin **1** is 0.73 μg/mL and IC_50_ for isoasarone **2** is 0.92 μg/mL, for *Ae*. *aegypti* which were not significantly different from *Ae*. *Albopictus*. The high inhibition of AChE and the fact that *P*. *sarmentosum* is used as food in local cuisine can bring wider spectrum to the use of asaricin **1** and isoasarone **2** as natural AChE inhibitors. The ever increasing emphasis on developing environmentally friendly pest control agents can brought these two compounds for further investigation. These results suggest that asaricin **1** and isoasarone **2** can be potential natural larvicide against *Ae*. *aegypti*, *Ae*. *albopictus and Cx*. *quinquefasciatus*. However, further research should be implemented such as stability and their impacts on human health and non-target organisms in mosquito feeding habitats.
